# The choice of surgical aortic valve replacement type and mid-term outcomes in 50 to 65-year-olds: results of the AUTHEARTVISIT study

**DOI:** 10.1093/ejcts/ezaf200

**Published:** 2025-06-17

**Authors:** Alissa Florian, Johann Auer, Berthold Reichardt, Pavla Krotka, Christine Wagenlechner, Ralph Wendt, Michael Mildner, Julia Mascherbauer, Hendrik Jan Ankersmit, Daniel Zimpfer, Alexandra Graf

**Affiliations:** Department of Cardiac Surgery, Medical University of Vienna, Vienna, Austria; Department of Internal Medicine I with Cardiology and Intensive Care, St Josef Hospital Braunau, Braunau am Inn, Austria; Austrian Social Health Insurance Fund, Eisenstadt, Austria; Center for Medical Data Science, Medical University of Vienna, Vienna, Austria; Center for Medical Data Science, Medical University of Vienna, Vienna, Austria; Department of Nephrology, St Georg Hospital, Leipzig, Germany; Department of Dermatology, Medical University of Vienna, Vienna, Austria; Department of Internal Medicine 3, University Hospital St Poelten, St Poelten, Austria; Karl Landsteiner University of Health Sciences, Krems an der Donau, ViennaAustria; Department of Thoracic Surgery, Medical University of Vienna, Vienna, Austria; Laboratory for Cardiac and Thoracic Diagnosis, Regeneration and Applied Immunology, Austria; Department of Cardiac Surgery, Medical University of Vienna, Vienna, Austria; Center for Medical Data Science, Medical University of Vienna, Vienna, Austria

**Keywords:** Aortic valve replacement, Mechanical prosthesis, Bioprosthesis

## Abstract

**OBJECTIVES:**

In recent years, the use of biological prosthetic valves has increased in patients under 65 years of age. This study evaluated overall survival, major adverse cardiac events and reoperation risk following surgical aortic valve replacement using either mechanical or biological prostheses in patients aged 50 to 65 years, aiming to provide data to support optimal valve selection in this group.

**METHODS:**

A registry-based cohort study was conducted using nationwide Austrian health insurance data from 1 January 2010 to 31 December 2020. Patients undergoing isolated surgical aortic valve replacement were classified based on valve type into mechanical or biological groups. The primary outcome was all-cause mortality. Secondary outcomes included major adverse cardiac events, reoperation, stroke, bleeding and survival after reoperation. Outcomes were assessed using Cox or competing risk regression models. Propensity score matching was used to reduce baseline differences.

**RESULTS:**

In the study cohort, 1018 patients were categorized to the mechanical and 2743 to the biological group. Patients who received mechanical valves had a significantly lower risk of death compared to those with biological valves (hazard ratio 1.352; *P* = 0.003). The biological group also had higher risks of major adverse cardiac events (hazard ratio 1.182; *P* = 0.03) and reoperation (hazard ratio 2.338; *P* = 0.002). Stroke and bleeding risks were similar between groups. All findings remained significant after propensity score matching.

**CONCLUSIONS:**

Among patients aged 50 to 65 years undergoing surgical aortic valve replacement, mechanical valves were associated with improved long-term survival, fewer major adverse events, and a lower need for repeat surgery. These findings suggest a need to re-evaluate the increasing use of biological valves in this age group.

## Graphical Abstract

**Figure:**
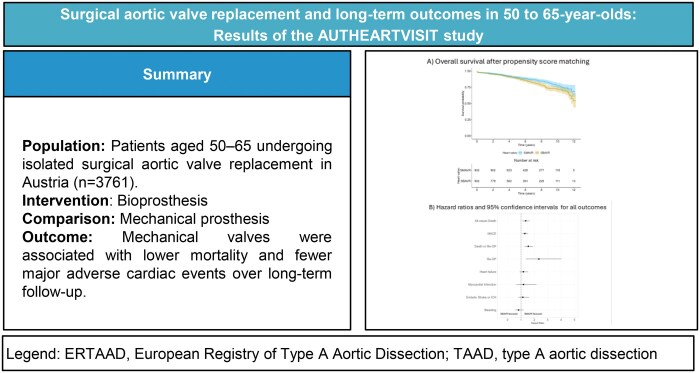

## INTRODUCTION

Valvular heart disease, particularly aortic valve stenosis, significantly impacts global cardiovascular morbidity and mortality [1, 2]. Symptomatic severe aortic valve disease is typically treated with aortic valve replacement (AVR) using biological or mechanical prostheses, homografts or autografts [3].

The European Society of Cardiology (ESC) guidelines recommend mechanical AVR (SMAVR) for patients ≤60 years and biological AVR (SBAVR) for those ≥65 years [4]. The American Heart Association (AHA)/American College of Cardiology (ACC) guidelines suggest sM-AVR for patients ≤50 years and sB-AVR for those ≥65 years [5].

Registry studies showing similar outcomes for both valve replacements [6–8] and higher stroke incidence after SMAVR [9] have influenced clinical practice and more frequent use of bioprostheses in younger patients. However, studies indicate increased mortality, major adverse cardiac events (MACEs) and reoperations after SBAVR [9–14]. Besides associating transcatheter aortic valve implantation (TAVI) in patients under 65 with higher all-cause-mortality [15], our group observed better long-term survival and lower reoperation risk after SMAVR in patients aged 50–65 [14] and ≤50 years [13].

Those discrepancies suggest the need for further evaluation to determine the optimal prosthesis for middle-aged patients. We conducted a cohort study of all patients in Austria who underwent isolated surgical AVR between 2010 and 2020, covered by the main Austrian insurers. Expanding on our previous study using the AUTHEARTVISIT registry, we included additional patients who had undergone surgery by 2020 and employed propensity score matching (PSM) for comparable groups. Real-life data based on ICD-10, ATC and MEL codes (see Supplementary Material, Tables S1–S5) were collected from Austrian insurance funds. The primary end-point was survival comparison after SBAVR or SMAVR, secondary end-points included MACEs, reoperations, survival after reoperation and stroke or bleeding after each procedure.

## PATIENTS AND METHODS

### Study design

In this nationwide, registry-based cohort study approved by the Ethics Committee of Lower Austria (GS1-EK-4/722–2021) and registered at clinicaltrials.gov (NCT06782620), in compliance with the Declaration of Helsinki, we obtained clinical and operative data for patients aged 50–65 years who underwent surgical AVR (SAVR) (SMAVR [MEL code DB082] or SBAVR [MEL codes DB060, DB070 and DB080]) between 1 January 2010 and 31 December 2020 in the Austrian healthcare system. The study data were generated retrospectively by retrieval from the Austrian Health Insurance Funds. Austria’s healthcare system operates as a national framework with broad access to medical care. The access to health services is regulated by social insurance law. All insured individuals have a legal entitlement to services. Austrian social insurance is founded on the principles of solidarity and self-administration, primarily financed through social insurance contributions. Around 98% of the Austrian population is enrolled in the public health insurance system. Therefore, only a small group of privately insured patients covering medical expenses could not be included. Consent was waived due to the receipt of anonymized data from the Insurance funds.

Patients who underwent TAVI (MEL codes DB025, DB026, DB021 or XN010) as index procedure, those aged <50 or >65 years, those with concomitant heart surgery or additional procedures during the index operation and patients receiving a coronary artery stent (MEL codes DD050 or DD060) within 4 months before AVR were excluded (see Supplementary Material, Section S1 and Fig. S1). We excluded patients undergoing multivalvular surgery or additional procedures performed during the index operation and patients receiving a coronary artery stent within 4 months prior to the AVR to guarantee the selection of patients with pure AVR procedures.

### Outcomes

The definition of outcomes is based on billing information (based on MEL codes) and diagnoses (based on ICD-10 codes) that were available from index operation date to the end of the study (details on coding see Supplementary Material, Tables S1–S3). The primary end-point was all-cause death based on the time from index-op to death-date or censoring. The secondary outcome reoperation was defined as the first event after index-op with MEL-codes as described in Table S3. Definitions of myocardial infarction, heart failure, embolic stroke or intracerebral haemorrhage (ICH) and bleeding other than ICH were based on ICD-10 codes (Table S2). MACE were defined as a combined end-point, that is, the first event after index-op including myocardial infarction, heart failure, embolic stroke or ICH, reoperation or death. We furthermore investigated the end-point death or reoperation (reoperation-free survival) as combined end-point (see Table S1). As an exploratory end-point, we evaluated the time from reoperation to death.

### Statistical analysis

Categorical variables are presented as counts and percentages, continuous variables as median and interquartile range (IQR).

The association between heart valve type and the primary outcome, time until death, was first evaluated using a multivariable cox proportional hazards model. This model was accounting for heart valve type (SMAVR or SBAVR), age, sex (Male or Female) and the following co-morbidities (binary variable: comorbidity present within 1 year before index surgery Yes or No): Myocardial Infarction, Embolic Stroke or ICH, Diabetes Mellitus, Adiposity, Hyperlipidaemia, Hyperuricaemia/Gout, Valvular-, rhythmological or other cardiomyopathies (CMPs), Ischaemic CMP, Artherosclerosis, Pulmonary diseases, Kidney diseases and Malignant diseases (for definitions see Table S5). Proportional hazard assumption was evaluated using Schönfeld residuals, collinearity was evaluated using variance inflation factors. Results of the multivariable Cox proportional hazard model are presented as hazard ratios and corresponding 95% confidence intervals as well as p-values. Survival probabilities (and corresponding 95% confidence intervals) were estimated using the Kaplan–Meier method. Follow-up times was calculated using the reverse Kaplan–Meier survival curve.

Due to a potential unbalance, we performed PSM between the 2 groups (SMAVR and SBAVR). The balance between heart valve groups before and after PSM is presented as standardized mean differences (SMD).

The propensity score was estimated with logistic regression based on age, sex and co-morbidities present 1 year before index surgery as listed for the original model. The matching was performed for a 1:1 ratio using a nearest-neighbour matching algorithm with a calliper width of 0.01 standard deviations for the logit of the propensity score. After propensity matching, mixed effect cox regression was used to account for PSM.

Secondary outcomes (MACEs and reoperation free survival) were analysed similarly to the primary end-point.

Time to remaining events was assessed using competing risk regression with death as competing event via the Fine and Gray method. Cumulative incidence functions were plotted, and results from competing risk regression models were provided as sub-distribution HRs with 95% CIs and p-values. After PSM, competing risk models including a clustering for the matching ID were performed.

Time to all-cause death after reoperation was evaluated descriptively using the Kaplan–Meier method due to limited events.

Models for heart failure and MACEs excluded patients with preoperative heart failure.

All analyses were performed using R version 4.3.2. For details on statistical analyses, see the Supplementary Material, Section S3. Statistical significance was set at *P* < 0.05. Due to the retrospective, exploratory nature of the study, no correction for multiplicity was applied; therefore, secondary and exploratory outcomes should be interpreted cautiously.

## RESULTS

### Study population and patient characteristics

**Figure 1:**
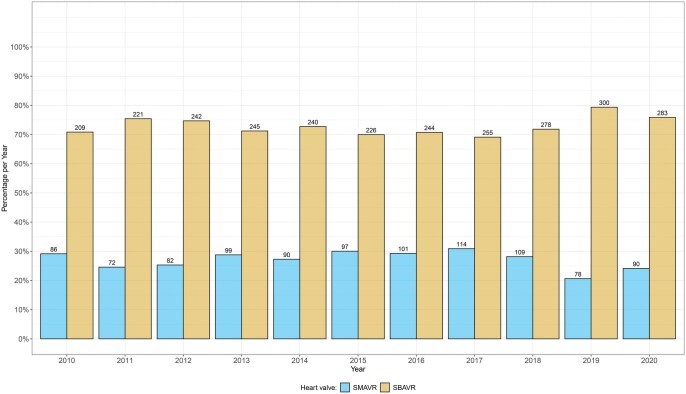
Percentages per year (y-axis) and absolute numbers (above bars) of patients receiving biological/mechanical valves.

From 1 January 2010 to 31 December 2020, 3761 patients underwent surgical AVR: 1018 SMAVR and 2743 SBAVR. Over all years, more patients underwent SBAVR (Fig. 1); however, there was no significant increasing trend over the years (*P* = 0.241). SBAVR patients were older with a higher incidence of diabetes mellitus, ischaemic CMP and malignant diseases, while hyperuricaemia/gout was more common in SMAVR patients (Table 1). For detailed medication information, see Supplementary Material, Table S6. Due to this group imbalance, we performed PSM (Table 1). Median follow-up was 6.20 years, 6.29 years in the SMAVR and 6.12 years in the SBAVR group.

**Table 1:** Characteristics and pre-existing medical diagnoses at time of index operation and standardized mean differences (SMDs) for description of balance between groups

	All data			After PSM		
Variables	SMAVR (*n* = 1018)	SBAVR (*n* = 2743)	SMD	SMAVR (*n* = 902)	SBAVR (*n* = 902)	SMD
Patients aged 50–65 years						
Median age, years (IQR)	56 (53–60)	61 (57–63)	−0.836	57 (54–60)	57 (54–60)	0.005
Sex, female	284 (27.9%)	743 (27.09%)	0.018	255 (28.27%)	233 (25.83%)	0.054
Heart failure	91 (8.94%)	295 (10.75%)	−0.064	74 (8.2%)	78 (8.65%)	−0.016
Myocardial infarction	24 (2.36%)	93 (3.39%)	−0.068	23 (2.55%)	18 (2%)	0.037
Embolic stroke or ICH	9 (0.88%)	48 (1.75%)	−0.092	9 (1%)	8 (0.89%)	0.012
Diabetes mellitus	118 (11.59%)	441 (16.08%)	−0.140	111 (12.31%)	105 (11.64%)	0.021
Obesity	84 (8.25%)	233 (8.49%)	−0.009	71 (7.87%)	62 (6.87%)	0.036
Hyperlipidaemia	254 (24.95%)	647 (23.59%)	0.032	207 (22.95%)	205 (22.73%)	0.005
Hyperuricaemia/gout	40 (3.93%)	71 (2.59%)	0.069	27 (2.99%)	28 (3.1%)	−0.006
Valvular, rhythmological, other CMPs	839 (82.42%)	2307 (84.1%)	−0.044	739 (81.93%)	735 (81.49%)	0.012
Ischaemic CMP	394 (38.7%)	1252 (45.64%)	−0.142	340 (37.69%)	357 (39.58%)	−0.039
Arteriosclerosis	33 (3.24%)	119 (4.34%)	0.062	31 (3.44%)	20 (2.22%)	0.069
Pulmonary diseases	31 (3.05%)	102 (3.72%)	−0.039	27 (2.99%)	25 (2.77%)	0.013
Kidney diseases	65 (6.39%)	194 (7.07%)	−0.028	57 (6.32%)	51 (5.65%)	0.027
Malignant diseases	16 (1.57%)	85 (3.1%)	−0.123	13 (1.44%)	15 (1.66%)	−0.018
Patients aged 50–60 years						
Median age, years (IQR)	55 (53–57)	57 (54–59)	−0.424	56 (53–58)	55 (53–58)	0.015
Sex, female	219 (27.07%)	290 (23.16%)	0.088	171 (25.37%)	172 (25.52%)	−0.003
Heart failure	61 (7.54%)	110 (8.79%)	−0.047	39 (5.79%)	47 (6.97%)	−0.045
Myocardial infarction	16 (1.98%)	36 (2.88%)	−0.064	12 (1.78%)	9 (1.34%)	0.032
Embolic stroke or ICH	6 (0.74%)	26 (2.08%)	−0.156	5 (0.74%)	4 (0.59%)	0.017
Diabetes mellitus	76 (9.39%)	161 (12.86%)	−0.119	58 (8.61%)	62 (9.2%)	−0.020
Obesity	65 (8.03%)	99 (7.91%)	0.005	43 (6.38%)	42 (6.23%)	0.005
Hyperlipidaemia	194 (23.98%)	265 (21.17%)	0.066	150 (22.26%)	132 (19.58%)	0.063
Hyperuricaemia/gout	28 (3.46%)	24 (1.92%)	0.084	16 (2.37%)	12 (1.78%)	0.032
Valvular, rhythmological, other CMPs	665 (82.2%)	1042 (83.23%)	−0.027	536 (79.53%)	546 (81.01%)	−0.039
Ischaemic CMP	292 (36.09%)	492 (39.3%)	−0.067	250 (37.09%)	241 (35.76%)	0.028
Arteriosclerosis	25 (3.09%)	40 (3.19%)	−0.006	20 (2.97%)	18 (2.67%)	0.017
Pulmonary diseases	20 (2.47%)	42 (3.35%)	−0.057	15 (2.23%)	17 (2.52%)	−0.019
Kidney diseases	46 (5.69%)	73 (5.83%)	−0.006	38 (5.64%)	30 (4.45%)	0.051
Malignant diseases	11 (1.36%)	34 (2.72%)	−0.117	10 (1.48%)	4 (0.59%)	0.077

Unless otherwise indicated, values are given as the number of patients with a diagnosis and corresponding percentage of total patients in the treatment group.

### Primary outcome

**Figure 2:**
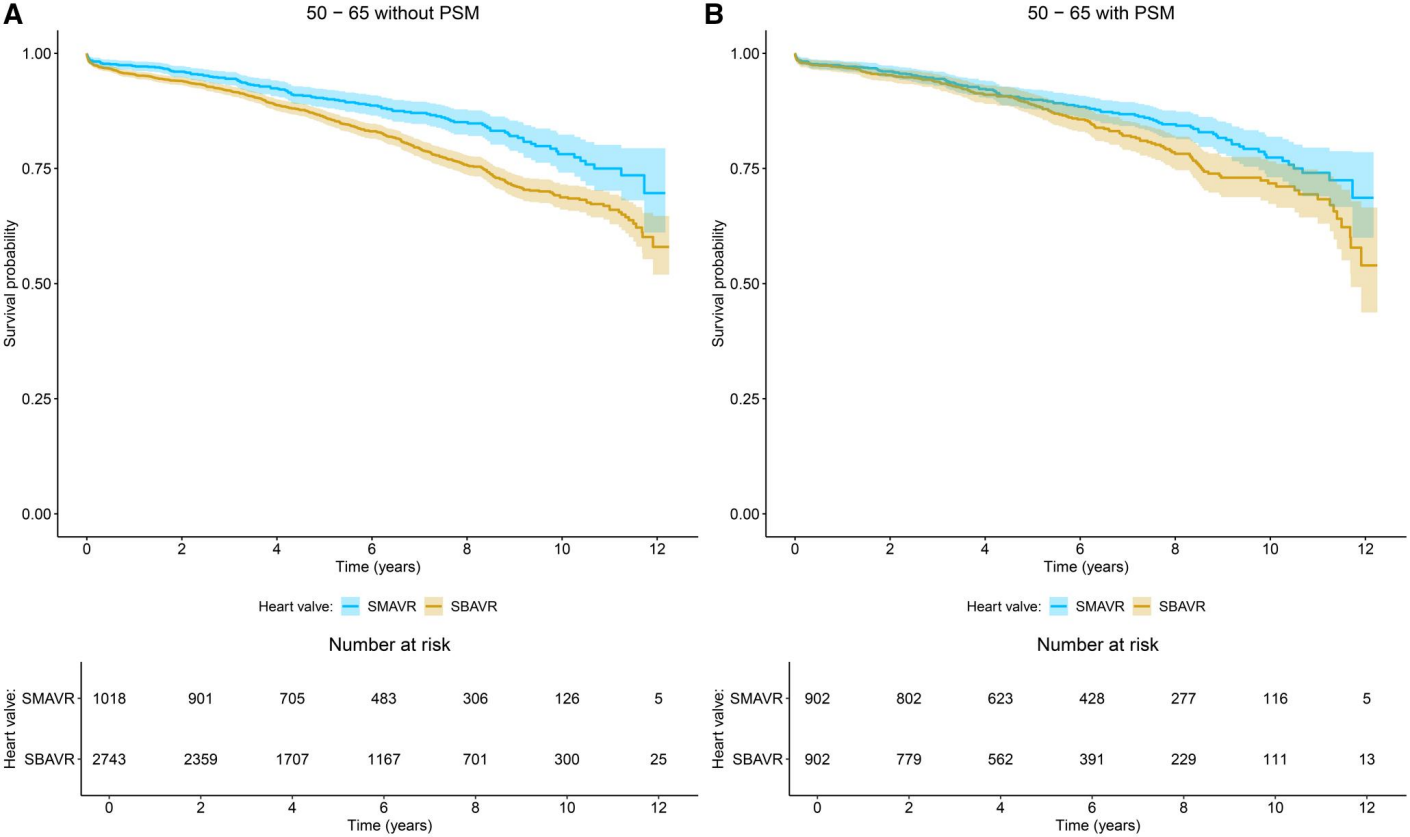
Kaplan–Meier curves and 95% confidence intervals for all-cause death before (**A**) and after (**B**) PSM. PSM: propensity score matching.

**Figure 3:**
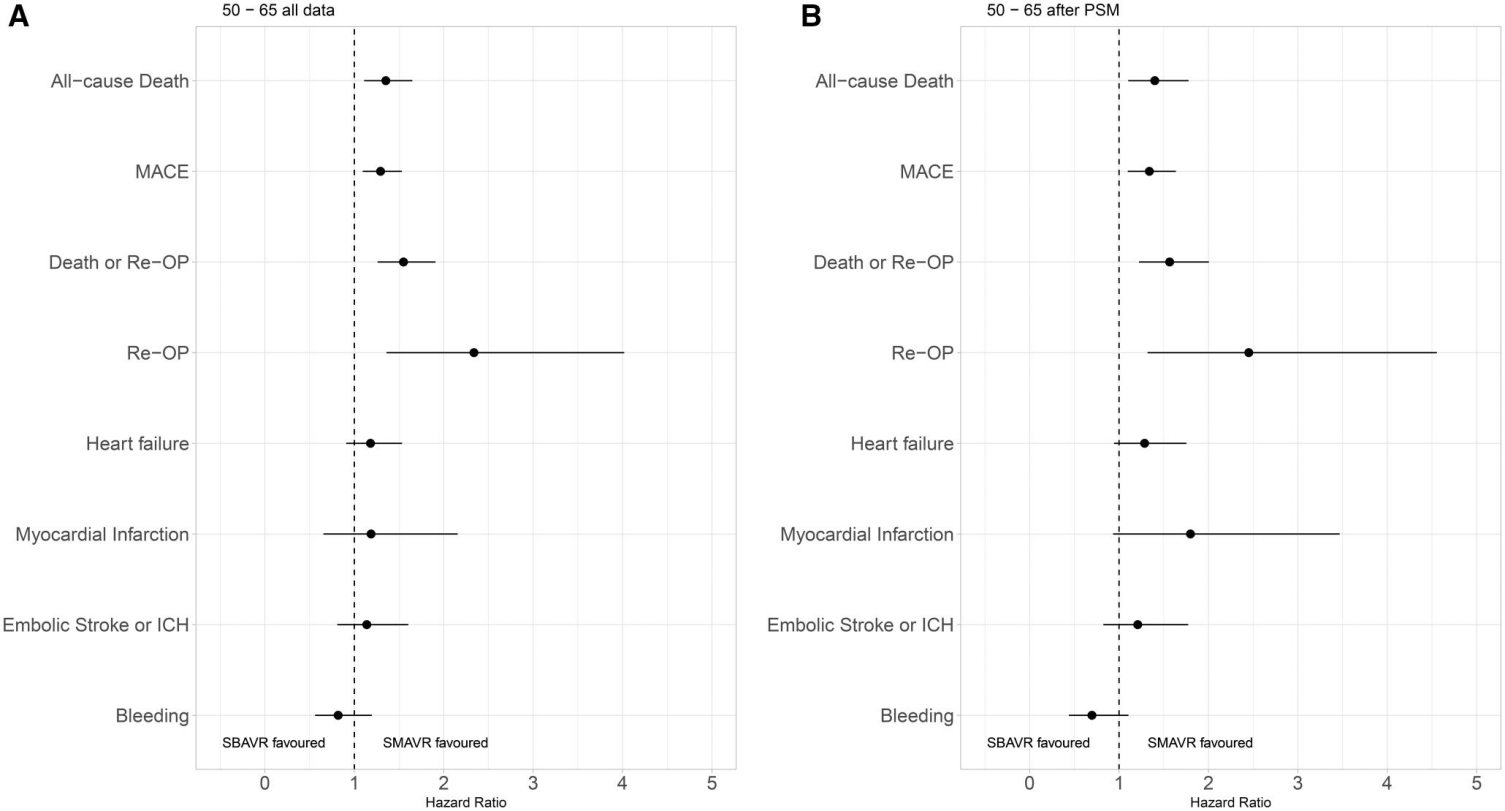
Hazard ratios (HRs) and 95% confidence intervals from multivariable regression models for all outcomes before (**A**) and after (**B**) PSM. HR >1 indicates a higher risk of the event occurring after SBAVR than SMAVR. PSM: propensity score matching; SBAVR: biological aortic valve replacement; SMAVR: mechanical aortic valve replacement.

SBAVR presented a higher risk of all-cause death than SMAVR (HR: 1.352; 95% CI: 1.109–1.649, *P* = 0.003, Figs 2 and 3A). PSM-analysis confirmed this (HR: 1.400; 95% CI: 1.103–1.778, *P* = 0.006, Figs 2 and 3B). For additional statistical model details and estimated survival probabilities, see Supplementary Material, Tables S7–S9. No significant tendency for varying treatment effects with increasing age was observed in the overall cohort or in the PSM-cohort (HR of interaction term of overall cohort: 0.965; 95%-CI: 0. 922–1.009, *P* = 0.118).

### Secondary outcomes

#### Major adverse cardiac events

A higher risk of MACEs was noted for SBAVR than for SMAVR (HR: 1.293; 95% CI: 1.093–1.531, *P* = 0.003, Fig. 3A), PSM did not alter this outcome (HR: 1.339; 95% CI: 1.097–1.636, *P* = 0.004, Fig. 3B, Supplementary Material, Tables S10–S12, Fig. S3).

##### Reoperation-free survival

Furthermore, a significantly lower probability of reoperation-free survival (see Supplementary Material, Tables S13–S15, Fig. S4) was observed for SBAVR compared to SMAVR (HR: 1.550; 95% CI: 1.260–1.908, *P* < 0.001, Fig. 3A). PSM did not change the results (HR: 1.566; 95% CI: 1.223–2.005, *P* < 0.001, Fig. 3B).

#### Reoperation

Concerning reoperation as outcome (see Supplementary Material, Tables S16–S18, Fig. S5), a significantly higher risk of reoperation was observed for SBAVR than SMAVR (sub-HR: 2.338; 95% CI: 1.360–4.019, *P* = 0.002, Fig. 3A). PSM did not change the results (sub-HR: 2.451; 95% CI: 1.319–4.555, *P* = 0.005, Fig. 3B).

##### Heart failure

We found no significant difference between SBAVR and SMAVR in the risk of newly diagnosed heart failure (Fig. 3, Supplementary Material, Tables S19–S21, Fig. S6) in our cohort (*P* = 0.21, after PSM: *P* = 0.11).

##### Myocardial infarction

We did not find a significant difference between SBAVR and SMAVR in the risk of myocardial infarction (Fig. 3, Supplementary Material, Tables S22–S24, Fig. S7) in our cohort (*P* = 0.57, after PSM: *P* = 0.08).

##### Embolic stroke and ICH

We found no significant difference between SBAVR and SMAVR in the risk of embolic stroke or ICH (Fig. 3, Supplementary Material, Tables S25–S27, Fig. S8) in the observed cohort (*P* = 0.46, after PSM: *P* = 0.33). Although the main group effect not being significant in the cohort aged 50–65 years, there was a trend indicating a decreasing advantage of SMAVR over SBAVR with increasing age (sub-HR: 0.905; 95%-CI: 0.846–0.968, *P* = 0.004). Post-PSM, the interaction between prosthesis type and age remained significant (*P* = 0.021).

#### Bleeding

We did not observe a significant association of the valve type (SBAVR and SMAVR) with the risk of bleeding (other than embolic stroke or ICH) in our cohort (*P* = 0.30; after PSM: *P* = 0.13, Fig. 3, Supplementary Material, Tables S28–S30, Fig. S9). However, in patients aged 50–65 years, there was a trend suggesting SMAVR might be advantageous for females but disadvantageous for males regarding bleeding (sub-HR for interaction term: 2.439, 95%-CI: 1.054–5.641, *P* = 0.037). Post-PSM, the interaction between prosthesis type and age remained significant (*P* = 0.045).

### Outcome after reoperation

Few events were observed regarding survival post-reoperation. The 5-year survival probabilities after reoperation were 85.2% (95% CI: 68–100%) and 75.8% (95% CI: 66.6–86.2%) for the SMAVR and the SBAVR group, respectively (see Supplementary Material, Table S31, Fig. S10).

## DISCUSSION

Middle-aged patients had significantly higher survival rates, lower risks of MACEs and reduced reoperation rates after SMAVR than SBAVR, aligning with prior research [16, 17].

Despite ESC guidelines recommending SMAVR for patients aged <60 [4], SBAVR was more common overall (72.93% vs 27.07%). It is noteworthy that over 9% of patients who eventually underwent SBAVR had been on oral anticoagulants in the year leading up to their surgery, even though the current ESC guidelines offer a Class IIb recommendation for mechanical valves in individuals already on long-term anticoagulation therapy (4.41% received vitamin K antagonists, and 4.7% received direct oral anticoagulants). After the SAVR, 92.7% of SMAVR patients received direct thrombin inhibitors, FXa inhibitors or Vitamin K antagonists while still 52.3% of SBAVR patients received those medications. AHA/ACC guidelines suggest SMAVR for those under 50 years and claim better haemodynamics and lower thromboembolic risk for SBAVR [5], but our data did not support these benefits. Contrary to previous studies [16], there was no significant difference in bleeding events after SMAVR.

Contrary to some studies [8, 9] but in line with the findings of Hammermeister, Diaz and Jiang [16, 18, 19], we found no significant difference in stroke incidence. In the PSM-cohort, the reoperation-risk was significantly higher after SBAVR than SMAVR. The 5-year survival probability post-reoperation was 85.2% for SMAVR and 75.8% for SBAVR, though based on limited events.

Most long-term survival data after SBAVR or SMAVR come from registry studies, such as Schnittman [8] and Goldstone [9]. Further studies found better long time-survival [11] and lower reoperation-risk [11, 18] after SMAVR, but some reported, contrary to our findings, higher major bleeding [11] or stroke-risk [19] after SMAVR.

Four meta-analyses by Diaz [20], Jiang [21], Warraich [22] and Leviner [23], associated SBAVR with worse survival, higher reoperation rates, and lower bleeding rates compared to SMAVR. Despite evidence of a survival benefit with SMAVR, recent guidelines and practices have favoured SBAVR, possibly due to avoiding lifelong anticoagulation and the potential for TAVI ‘valve-in-valve’ options. Chiang *et al.* [6] underscored that complications associated with mechanical valves can be more severe and present fewer minimally invasive rescue options compared to the deterioration of biological valves. This underscores the significance of considering both the frequency of outcomes and the manageability of complications when selecting a prosthesis. This debate prompted us to expand our patient cohort from 2612 to 3824 patients, with a median follow-up of 6.2 years up to a maximum of 12 years, overcoming previous study limitations. We performed PSM and included stroke, ICH and general bleeding in our analysis to create comparable groups, addressing key prosthesis choice arguments [14].

Registry studies and meta-analyses primarily compare survival, reoperation rates and MACEs. Percy *et al.* proved that over 40% of patients under 65 years develop subclinical structural valve degeneration (SVD), with 21.5% progressing to clinical SVD or requiring repeat procedures as early as 11 months after SBAVR [24]. Salaun *et al.* found that after a median follow-up of 6.7 months, 25.6% of patients had leaflet calcifications on CT scans and 38% developed haemodynamic valve deterioration [25]. Studies have shown that age-dependent accelerated SVD after SBAVR increases reoperation likelihood, especially in younger patients [26–28]. Therefore, the selection of an aortic valve prosthesis requires careful consideration. Additionally, the possibility of younger patients rejecting their bioprostheses due to humoral and cellular immune responses must be considered [29–31].

### Limitations

We utilized real-world data from Austrian insurance funds, acknowledging observational research limitations. Data were collected from all patients in the Austrian Health System who underwent SAVR from 1 January 2010 to 31 December 2020. With Austria’s extensive healthcare coverage, about 98% of the population is registered.

Although our retrospective data lacked the rigor of prospective randomized trials and controlled treatment allocation, we aimed to create comparable patient groups using PSM, reducing bias that SMAVR might be performed more often than SBAVR in younger, healthier individuals.

The administrative data from billing records and discharge codes depend on accurate nationwide coding of diseases and events. We cannot retrospectively verify or correct these data, potentially introducing bias and inconsistencies compared to prospective clinical databases. Additionally, we could not examine medical reasons for death or reoperation, preventing the exclusion of patients with outcomes unrelated to the original surgery. However, our outcome measures involved hospital-required events likely to be accurately reported to Austrian health insurance carriers for billing.

## CONCLUSIONS

Our data indicate that the trend of lowering the age limits for SBAVR requires critical reassessment. We observed significantly higher mortality and reoperation risks in younger patients after SBAVR. Thus, selection of aortic valve prostheses in younger patients should be approached with caution.

## Supplementary Material

ezaf200_Supplementary_Data

## Data Availability

Due to data protection, the datasets presented in this article are not readily available. Data that support the findings of this study are available upon reasonable request from the corresponding author.

## ABBREVIATIONS

AVR: Aortic valve replacement
CMP: Cardiomyopathy
ICH: Intracerebral haemorrhage
MACE: Major adverse cardiac event
PSM: Propensity score matching
SAVR: Surgical aortic valve replacement
SBAVR: Biological aortic valve replacement
SMAVR: Mechanical aortic valve replacement
SVD: Structural valve degeneration
TAVI: Transcatheter aortic valve implantation
