# Supplementary material for: The choice of surgical aortic valve replacement type and mid-term outcomes in 50 to 65-year-olds: results of the AUTHEARTVISIT study
Source: Eur J Cardiothorac Surg. 2025 Jun 17;67(7):ezaf200. doi: 10.1093/ejcts/ezaf200 (PMC12231540; doi:10.1093/ejcts/ezaf200)

Supplemental Material to The choice of surgical aortic valve replacement type and mid-term outcomes in 50 to 65-year-olds: Results of the AUTHEARTVISIT study

A. Florian[1], J. Auer[2], B. Reichardt[3], P. Krotka[4], C. Wagenlechner[4], R. Wendt[5], M. Mildner[6], J. Mascherbauer[7,8], HJ Ankersmit[9,10*#], D. Zimpfer[1*#], A. Graf[4#]

1 Department of Cardiac Surgery, Medical University of Vienna, Austria
2 Department of Internal Medicine I with Cardiology and Intensive Care, St. Josef Hospital Braunau, Braunau am Inn, Austria
3 Austrian Social Health Insurance Fund, Eisenstadt, Austria
4 Center for Medical Data Science, Medical University of Vienna, Austria
5 Department of Nephrology, St. Georg Hospital, Leipzig, Germany
6 Department of Dermatology, Medical University of Vienna, Austria
7 Department of Internal Medicine 3, University Hospital St. Poelten, St. Poelten, Austria
8 Karl Landsteiner University of Health Sciences, Krems an der Donau, Austria
9 Department of Thoracic Surgery, Medical University of Vienna, Austria
10 Laboratory for Cardiac and Thoracic Diagnosis, Regeneration and Applied Immunology, Austria
# contributed equally
* corresponding authors

1.) General information on the cohort analyzed

This retrospective national registry-based study complied with the Declaration of Helsinki and was approved by the ethics committee of lower Austria (GS1-EK-4/722-2021). The trial was registered with ClinicalTrials.gov (NCT05912660). Study data were generated retrospectively by retrieval from the Austrian Health Insurance Funds. Data on outcomes and potential confounding factors were derived from billing information based on MEL (i.e., Medizinische Einzelleistung, or individual medical procedure) and International Classification of Diseases (ICD) codes available for each patient from one year before surgery up to study cut-off.

Austria's health care system operates as a national framework with broad access to medical care. The access to health services is regulated by social insurance law. All insured individuals have a legal entitlement to services. Austrian social insurance is founded on the principles of solidarity and self-administration, primarily financed through social insurance contributions. Around 98% of the Austrian population is enrolled in the public health insurance system. Therefore, only a small group of privately insured patients covering medical expenses could not be included in the AUTHEART-VISIT-Study. A flow chart of in- and excluded patients is shown in Figure 1.

1.1. Inclusion Criteria

For the presented analyses of the AUTHEARTVISIT study, clinical and operative data were obtained for all patients registered in the Austrian Health Care System who underwent surgical aortic valve replacement (SAVR) using a mechanical prosthesis (SMAVR, MEL code DB082 [Replacement of aortic valve with artificial mechanical valve]) or biological prosthesis (SBAVR, MEL codes DB060 [Replacement of aortic valve with pulmonary autograft], DB070 [Replacement of aortic valve with stentless valve], and DB080 [Replacement of aortic valve with stented valve]) in Austria from 01.01.2010 to 31.12.2020 and were aged between 50 and 65 years.

1.2. Exclusion Criteria

Patients receiving transcatheter aortic valve implantation (TAVI; MEL codes DB025 [Aortic valve replacement – catheter directed, transapical, TAVR], DB026 [Aortic valve replacement – catheter directed, transvalvular, TAVR], DB021 [Aortic valve replacement – percutaneous, interventional, TAVR], or XN010 [Aortic valve replacement – percutaneous, interventional, TAVR]) as the index operation were excluded from the sample.

Patients aged <50 years and >65 years were excluded from the data.

Patients with concomitant heart surgery were excluded from the data, i.e. patients with at least one valve surgery in addition to the index operation (SMAVR or SBAVR) on the date of the index operation.

Patients receiving a coronary artery stent (MEL code DD050 [implantation of a stent in the coronary artery] or DD060 [implantation of a drug eluting stent in a coronary artery]) within 4 months prior to the Index-SAVR were excluded from the analyses.

Note that we excluded patients undergoing multivalvular surgery or additional procedures performed during the index operation and patients receiving a coronary artery stent within 4 months prior to the AVR to guarantee the selection of patients with pure AVR procedures.

Figure S1: Flow-diagram to describe analyzed cohorts

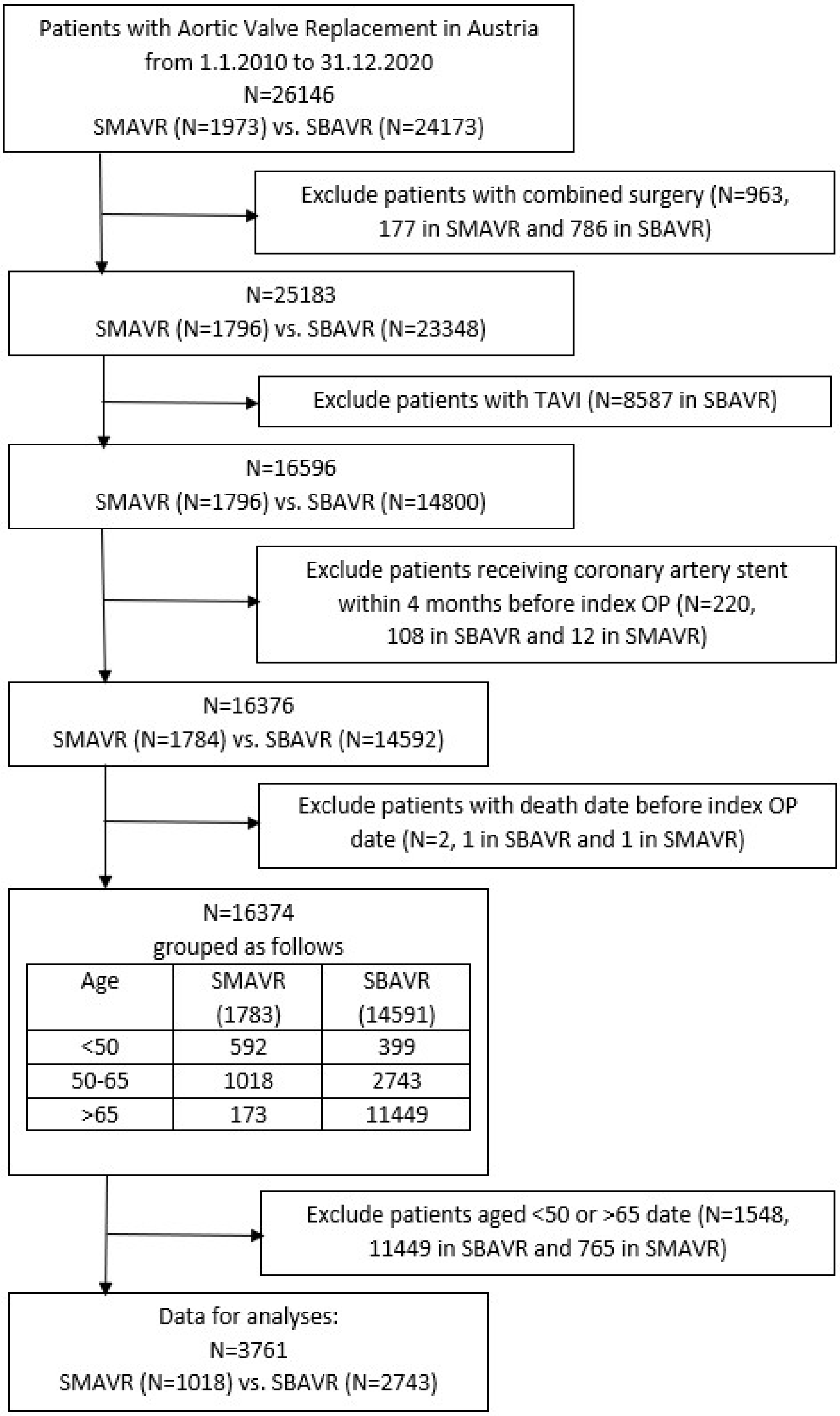

2.) Definition of Outcomes

For each patient, billing information (based on MEL codes from the Austrian insurance carriers) and diagnoses (based on ICD-10 codes) were available from 1 year before the index operation to the end of the study. To evaluate the diagnoses, we used the 10th revision of the International Statistical Classification of Diseases and Related Health Problems (ICD-10 from 2019), which is available at

https://icd.who.int/browse10/2019/en

The corresponding German version is available at

https://www.dimdi.de/static/de/klassifikationen/icd/icd-10-who/kode-suche/htmlamtl2019/.

For each patient, billing information (based on MEL codes) and diagnoses (based on ICD-10 codes) were available from 1 year before the index operation to the end of the study. Death dates were available until the end of the study. To evaluate the different outcomes for each patient, data were scanned for the corresponding codes from the index operation to the end of the study as shown in the following tables. Index operation (Index-OP) was either SMAVR or SBAVR as described in the Inclusion Criteria (1.1).

**Outcome**	**Definition**
**Primary**	
All-cause death	All-cause death based on death date (time from Index-OP to death)
**Secondary**	
MACE	Major adverse cardiac event defined as combined endpoint: time from Index-OP to the first event after Index-OP with ICD codes (Table S2) for myocardial infarction, heart failure, embolic stroke or ICH, reoperation, or death based on death date
Death or reoperation	Combined endpoint: time from Index-OP to the first event after Index-OP with MEL code defined as in Table S3 or death based on death date
Reoperation	Based on billing information (MEL code): time from Index-OP to the first event after Index-OP on with MEL code defined as in Table S3
Myocardial infarction	Based on ICD codes: time from the Index-OP to the first event after Index-OP with ICD code for Myocardial infarction defined as in Table S2
Heart failure	Based on ICD codes: time from Index-OP to the first event after Index-OP with ICD code for Heart failure defined as in Table S2
Embolic stroke or intracerebral hemorrhage (ICH)	Based on ICD codes: time from Index-OP to the first event after Index-OP with ICD code for Embolic stroke or ICH defined as in Table S2
Bleeding other than ICH	Based on ICD codes: time from Index-OP to the first event after Index-OP with ICD code for Bleeding other than ICH defined as in Table S2
**Exploratory**	
Death after reoperation	Time from first reoperation date after Index-OP (defined as in Table S3) to death date

Table S1: Definitions of outcomes

Outcome	ICD-10 Codes
Myocardial infarction	I21.0, I21.1, I21.2, I21.3, I21.4, I21.9
Heart failure	I11.0, I13.0, I13.2, I50.0, I50.1, I50.9, I50.11, I50.12, I50.13, I50.14, I50.19
Embolic stroke or intracerebral hemorrhage (ICH)	I63.0, I63.1, I63.2, I63.3, I63.4, I63.5, I63.6, I63.8, I63.9, G45.9, G45.0, G45.1, G45.2, G45.3, G45.4, G45.8, I61.0, I61.1, I61.2, I61.3, I61.4, I61.5, I61.6, I61.8, I61.9, I64
Bleeding other than ICH	I60.0, I60.1, I60.2, I60.3, I60.4, I60.5, I60.6, I60.7, I60.8, I60.9, I85.0, I98.2, I98.3, K25.0, K25.1, K25.2, K25.3, K25.4, K25.5, K25.6, K25.7, K25.9, K26.0, K26.1, K26.2, K26.3, K26.4, K26.5, K26.6, K26.7, K26.9, K27.0, K27.1, K27.2, K27.3, K27.4, K27.5, K27.6, K27.7, K27.9, K28.0, K28.1, K28.2, K28.3, K28.4, K28.5, K28.6, K28.7, K28.9, K29.0, K29.1, K29.2, K29.3, K29.4, K29.5, K29.6, K92.2, N42.1, R04.1, R04.8, R04.9, R58, S06.4, T81.0

Table S2: ICD-10 codes for the definition of outcomes

MEL Code	Description
DB020	Percutaneous implantation of a pulmonary valve
DB025	Aortic valve replacement – catheter directed, transapical, TAVR
DB026	Aortic valve replacement – catheter directed, transvalvular, TAVR
DB030	Reconstruction of the aortic valve
DB040	Reconstruction of the mitral valve
DB050	Reconstruction of the tricuspid valve
DB055	Reconstruction of the pulmonary valve
DB060	Replacement of aortic valve with pulmonary autograft
DB070	Replacement of aortic valve with stentless valve
DB080	Replacement of aortic valve with stented valve
DB082	Replacement of aortic valve with artificial mechanical valve
DB090	Replacement of mitral valve with stentless valve
DB100	Replacement of mitral valve with stented valve
DB102	Replacement of mitral valve with artificial mechanical valve
DB110	Replacement of tricuspid valve with stentless valve
DB120	Replacement of tricuspid valve with stented valve
DB122	Replacement of tricuspid valve with artificial mechanical valve
DB130	Replacement of pulmonary valve with stentless valve
DB140	Replacement of pulmonary valve with stented valve
DB142	Replacement of pulmonary valve with artificial mechanical valve
DB021	Aortic valve replacement – percutaneous, interventional, TAVR
XN010	Aortic valve replacement – percutaneous, interventional, TAVR

Table S3: MEL-codes for the definition of the outcome reoperation

3.) Definitions of Confounders/Comorbidities

The index operation (Index-OP) group was defined using billing information based on MEL codes as in Table S4.

MEL Code	Description	Group
DB060	Replacement of aortic valve with pulmonary autograft	SBAVR
DB070	Replacement of aortic valve with stentless valve	SBAVR
DB080	Replacement of aortic valve with stented valve	SBAVR
DB082	Replacement of aortic valve with artificial mechanical valve	SMAVR

Table S4: Coding for prosthesis variable. SBAVR = bioprosthesis; SMAVR = mechanical aortic valve replacement

Comorbidities were defined using ICD-10 codes available for each patient up to 1 year before the index operation. Data available 1 year before the index operation were scanned for each patient based on the following ICD-10 codes, categorized for different comorbidities (Table S5). If at least once during the year prior to the index operation an ICD-10 code for a comorbidity was observed as the main or secondary diagnosis, the patient was assumed to suffer from this comorbidity.

Comorbidity	ICD Codes
Diabetes mellitus	E10.0, E10.1, E10.2, E10.3, E10.4, E10.5, E10.6, E10.7, E10.8, E10.9, E11.0, E11.1, E11.2, E11.3, E11.4, E11.5, E11.6, E11.7, E11.8, E11.9, E12.0, E12.1, E12.2, E12.3, E12.4, E12.5, E12.6, E12.7, E12.8, E12.9, E13.0, E13.1, E13.2, E13.3, E13.4, E13.5, E13.6, E13.7, E13.8, E13.9, E14.0, E14.1, E14.2, E14.3, E14.4, E14.5, E14.6, E14.7, E14.8, E14.9
Adiposity	E65, E66.0, E66.1, E66.2, E66.8, E66.9
Hyperlipidemia	E78.0, E78.1, E78.2, E78.3, E78.4, E78.5, E78.6, E78.8, E78.9
Hyperuricemia/gout	E79.0, E79.8, M10.0, M10.00, M10.01, M10.02, M10.03, M10.04, M10.05, M10.06, M10.07, M10.08, M10.09
Valvular, rhythmological, and other cardiomyopathies (CMPs)	I01.0, I01.1, I01.2, I01.8, I01.9, I02.0, I02.9, I05.0, I05.1, I05.2, I05.8, I05.9, I06.0, I06.1, I06.2, I06.8, I06.9, I07.0, I07.1, I07.2, I07.8, I07.9, I08.0, I08.1, I08.2, I08.3, I08.8, I08.9, I09.0, I09.1, I09.2, I09.8, I09.9, I10, I11.0, I11.9, I12.0, I12.9, I13.0, I13.1, I13.2, I13.9, I15.0, I15.1, I15.2, I15.8, I15.9, I26.0, I26.9, I27.0, I27.1, I27.2, I27.8, I27.9, I28.0, I28.1, I28.8, I28.9, I30.0, I30.1, I30.8, I30.9, I31.0, I31.1, I31.2, I31.3, I31.8, I31.9, I32.0, I32.1, I32.8, I33.0, I33.9, I34.0, I34.1, I34.2, I34.8, I34.9, I35.0, I35.1, I35.2, I35.8, I35.9, I36.0, I36.1, I36.2, I36.8, I36.9, I37.0, I37.1, I37.2, I37.8, I37.9, I38, I39.0, I39.1, I39.2, I39.3, I39.4, I39.8, I40.0, I40.1, I40.8, I40.9, I41.0, I41.1, I41.2, I41.8, I42.0, I42.1, I42.2, I42.3, I42.4, I42.5, I42.6, I42.7, I42.8, I42.9, I43.0, I43.1, I43.2, I43.8, I44.0, I44.1, I44.2, I44.3, I44.4, I44.5, I44.6, I44.7, I45.0, I45.1, I45.2, I45.3, I45.4, I45.5, I45.6, I45.8, I45.9, I46.0, I46.1, I46.9, I470., I47.1, I47.2, I47.9, I48.0, I48.1, I48.2, I48.3, I48.4, I48.9, I49.0, I49.1, I49.2, I49.3, I49.4, I49.5, I49.8, I49.9, I50.0, I50.11, I50.12, I50.13, I50.14, I50.19, I50.9, I51.0, I51.1, I51.2, I51.3, I51.4, I51.5, I51.6, I51.7, I51.8, I51.9, I52.0, I52.1, I52.8, Q20.0, Q20.1, Q20.2, Q20.3, Q20.4, Q20.5, Q20.6, Q20.8, Q20.9, Q21.0, Q21.1, Q21.2, Q21.3, Q21.4, Q21.8, Q21.9, Q22.0, Q22.1, Q22.2, Q22.3, Q22.4, Q22.5, Q22.6, Q22.8, Q22.9, Q23.0, Q23.1, Q23.2, Q23.3, Q23.4, Q23.8, Q23.9, Q24.0, Q24.1, Q24.2, Q24.3, Q24.4, Q24.5, Q24.6, Q24.8, Q24.9, Q25.0, Q25.1, Q25.2, Q25.3, Q25.4, Q25.5, Q25.6, Q25.7, Q25.8, Q25.9
Atherosclerosis	I69.8, I70.0, I70.1, I70.2, I70.8, I70.9
Pulmonary disease	J43.1, J43.2, J43.8, J43.9, J44.00, J44.01, J44.02, J44.03, J44.09, J44.10, J44.11, J44.12, J44.13, J44.19, J44.80, J44.81, J44.82, J44.83, J44.89, J44.90, J44.91, J44.92, J44.93, J44.99, J45.0, J45.1, J45.8, J45.9
Kidney disease	N00,0, N00.1, N00.2, N00.3, N00.4, N00.5, N00.6, N00.7, N00.8, N00.9, N01.0, N01.1, N01.2, N01.3, N01.4, N01.5, N01.6, N01.7, N01.8, N01.9, N02.0, N02.1,

	N02.2, N02.3, N02.4, N02.5, N02.6, N02.7, N02.8, N02.9, N03.0, N03.1, N03.2, N03.3, N03.4, N03.5, N03.6, N03.7, N03.8, N03.9, N04.0, N04.1, N04.2, N04.3, N04.4, N04.5, N04.6, N04.7, N04.8, N04.9, N05.0, N05.1, N05.2, N05.3, N05.4, N05.5, N05.6, N05.7, N05.8, N05.9, N06.0, N06.1, N06.2, N06.3, N06.4, N06.5, N06.6, N06.7, N06.8, N06.9, N07.0, N07.1, N07.2, N07.3, N07.4, N07.5, N07.6, N07.7, N07.8, N07.9, N08.0, N08.1, N08.2, N08.3, N08.4, N08.5, N08.8, N10, N11.0, N11.1, N11.8, N11.9, N12, N13.0, N13.1, N13.2, N13.3, N13.4, N13.5, N13.6, N13.7, N13.8, N13.9, N14.0, N14.1, N14.2, N14.3, N14.4, N15.0, N15.1, N15.8, N15.9, N16.0, N16.1, N16.2, N16.3, N16.4, N16.5, N16.8, N17.0, N17.1, N17.2, N17.8, N17.9, N18.1, N18.2, N18.3, N18.4, N18.5, N18.9, N19, N20.0
Ischemic cardiomyopathies (CMP)	I20.0, I20.1, I20.8, I20.9, I21.0, I21.1, I21.2, I21.3, I21.4, I21.9, I22.0, I22.1, I22.8, I22.9, I23.0, I23.1, I23.2, I23.3, I23.4, I23.5, I23.6, I23.8, I24.0, I24.1, I24.8, I24.9, I25.0, I25.1, I25.2, I25.3, I25.4, I25.5, I25.6, I25.8, I25.9
Malignant diseases	C00.0, C00.1, C00.2, C00.3, C00.4, C00.5, C00.6, C00.8, C00.9, C01, C02.0, C02.1, C02.2, C02.3, C02.4, C02.8, C02.9, C03.0, C03.1, C03.9, C04.0, C04.1, C04.8, C04.9, C05.0, C05.1, C05.2, C05.8, C05.9, C06.0, C06.1, C06.2, C06.8, C06.9, C07 , C08.0, C08.1, C08.8, C08.9, C09.0, C09.1, C09.8, C09.9, C10.0, C10.1, C102., C10.3, C10.4, C10.8, C10.9, C11.0, C11.1, C11.2, C11.3, C11.8, C11.9, C12, C13.0, C13.1, C13.2, C13.8, C13.9, C14.0, C14.2, C14.8, C15.0, C15.1, C15.2, C15.3, C15.4, C15.5, C15.8, C15.9, C16.0, C16.1, C16.2, C16.3, C16.4, C16.5, C16.6, C16.8, C16.9, C17.0, C17.1, C17.2, C17.3, C17.8, C17.9, C18.0, C180.1, C18.02, C180.3, C18.04, C18.1, C18.11, C18.12, C18.13, C18.14, C182., C18.21, C18.22, C18.23, C18.24, C18.3, C18.31, C18.32, C18.33, C18.34, C18.4, C18.41, C18.42, C18.43, C18.44, C18.5, C18.51, C18.52, C18.53, C18.54, C18.6, C18.61, C18.62, C18.63, C18.64, C18.7, C18.71, C18.72, C18.73, C18.74, C18.8, C18.81, C18.82, C18.83, C18.84, C18.9, C18.91, C18.92, C18.93, C18.94, C19, C19.1, C19.2, C19.3, C19.4, C20, C20.1, C20.2, C20.3, C20.4, C21.0, C21.1, C21.2, C21.8, C22.0, C22.1, C22.2, C22.3, C22.4, C22.7, C22.9, C23, C24.0, C24.1, C24.8, C24.9, C25.0, C25.1, C25.2, C25.3, C25.4, C25.7, C25.8, C25.9, C26.0, C26.1, C26.8, C26.9, C30.0, C30.1, C31.0, C31.1, C31.2, C31.3, C31.8, C31.9, C32.0, C32.1, C32.2, C32.3, C32.8, C32.9, C33, C34.0, C34.1, C34.2, C34.3, C34.8, C34.9, C37, C38.0, C38.1, C38.2, C38.3, C38.4, C38.8, C39.0, C39.8, C39.9, C40.0, C40.1, C40.2, C40.3, C40.8, C40.9, C41.0, C41.1, C41.2, C41.3, C41.4, C41.8, C41.9, C43.0, C43.1, C43.2, C43.3, C43.4, C43.5, C43.6, C43.7, C43.8, C43.9, C44.0, C44.1, C44.2, C44.3, C44.4, C44.5, C44.6, C44.7, C44.8, C44.9, C45.0, C45.1, C45.2, C45.7, C45.9, C46.0, C46.1, C46.2, C46.3, C46.7, C46.8, C46.9, C47.0, C47.1, C47.2, C47.3, C47.4, C47.5, C47.6, C47.8, C47.9, C48.0, C48.1, C48.2, C48.8, C49.0, C49.1, C49.2, C49.3, C49.4, C49.5, C49.6, C49.8, C49.9, C50.0, C50.1, C50.2, C50.3, C50.4, C50.5, C50.6, C50.8, C50.9, C51.0, C51.1, C51.2, C51.8, C51.9, C52, C53.0, C53.1, C53.8, C53.9, C54.0, C54.1, C54.2, C54.3, C54.8, C54.9, C55, C56, C57.0, C57.1, C57.2, C57.3, C57.4, C57.7, C57.8, C57.9, C58, C60.0, C60.1, C60.2, C60.8, C609., C61, C62.0, C62.1, C62.9, C63.0, C63.1, C63.2, C63.7, C63.8, C63.9, C64, C65, C66, C67.0, C67.1, C67.2, C67.3, C67.4, C67.5, C67.6, C67.7, C67.8, C67.9, C68.0, C68.1, C68.8, C68.9, C69.0, C69.1, C69.2, C69.3, C69.4, C69.5, C69.6, C69.8, C69.9, C70.0, C70.1, C70.9, C71.0, C71.1, C71.2, C71.3, C71.4, C71.5, C71.6, C71.7, C71.8, C71.9, C72.0, C72.1, C72.2, C72.3, C72.4, C72.5, C72.8, C72.9, C73, C740., C74.1, C749., C75.0, C75.1, C75.2, C75.3, C75.4, C75.5, C75.8, C75.9, C76.0, C76.1, C76.2, C76.3, C76.4, C76.5, C76.7, C76.8, C77.0, C77.1, C77.2, C77.3, C77.4, C77.5, C77.8, C77.9, C78.0, C78.1, C78.2, C78.3, C78.4, C78.5, C78.6, C78.7, C78.8, C79.0, C79.1, C79.2, C79.3, C79.4, C79.5, C79.6, C79.7, C79.8, C79.9, C80.0, C80.9, C81.0, C81.1, C81.2, C81.3, C81.4, C81.7, C81.9, C82.0, C82.1, C82.2, C82.3, C82.4, C82.5, C82.6, C82.7, C82.9, C83.0, C83.1, C83.3, C83.5, C83.7, C83.8, C83.9, C840., C84.1, C84.4, C845., C84.6, C84.7, C84.8, C84.9, C85.1, C85.2, C85.7, C85.9, C86.0, C86.1, C86.2, C86.3, C86.4, C86.5, C86.6, C88.0, C88.2, C88.3, C88.4, C887., C88.9, C90.0, C90.1, C90.2, C90.3, C91.0, C91.1, C91.3, C91.4, C91.5, C91.6, C91.7, C91.8, C91.9, C92.0, C92.1, C92.2, C92.3, C92.4, C92.5, C92.6,

	C92.7, C92.8, C92.9, C93.0, C93.1, C93.3, C93.7, C93.9, C94.0, C94.2, C94.3, C94.4, C94.6, C94.7, C95.0, C95.1, C95.7, C95.9, C96.0, C96.2, C96.4, C96.5, C96.6, C96.7, C96.8, C96.9, C97
Stroke before OP	ICD-Codes as in Table S2
Heart failure before OP	ICD-Codes as in Table S2
Heart attack before OP	ICD-Codes as in Table S2

Table S5: ICD-10 codes for comorbidities

3.) Statistical Analyses

The goal of the study was to evaluate the association between the heart valve type (SMAVR or SBAVR, see Table S4) and the following endpoints (detailed definitions see Table S1 – S3):

Primary Outcome:
- Time until all-cause death

Secondary Outcomes:
- Time until the combined endpoint major adverse cardiac event (MACE)
- Time until the combined endpoint death or reoperation
- Time until reoperation
- Time until heart failure
- Time until myocardial infarction
- Time until embolic stroke or ICH
- Time until bleeding other than embolic stroke or ICH

Exploratory Outcome:
- Time to all-cause death after reoperation

All p-values smaller than 0.05 were considered as statistically significant. Due to the retrospective and exploratory character of the study no correction for multiplicity was applied. Results on secondary and exploratory outcomes may therefore be handled with care.

Categorical variables are shown as counts and percentages, while continuous variables are summarized using medians as well as 1st and 3rd quartiles. Chi-square tests for categorical data and t-tests for age were performed to compare baseline characteristics between groups (SMAVR, SBAVR). A Cochran-Armitage Trend test was performed to evaluate the trend of SBAVR use over the years.

3.1. Analyses of primary outcome

The association between heart valve type and the primary outcome, time until death, was first evaluated using a multivariable cox proportional hazards model. This model was accounting for heart valve type (SMAVR or SBAVR), age, sex (Male or Female) and the following co-morbidities (binary variable: comorbidity present within one year before index surgery Yes or No): Myocaridal Infarction, Embolic Stroke or ICH, Diabetes Mellitus, Adiposity, Hyperlipidemia, Hyperuricemia/Gout, Valvular-, rhythmological or other CMPs, Ischemic CMP, Artherosclerosis, Pulmonary diseases, Kidney diseases and Malignant diseases (definitions see Table S5). Proportional hazard assumption was evaluated using Schönfeld residuals, collinearity was evaluated using variance inflation factors. Results of the multivariable Cox proportional hazard model are presented as hazard ratios and corresponding 95% confidence intervals as well as p-values. Significant hazard ratios larger than one for the predictor "heart valve type" are to be interpreted as follows: patients with SBAVR valve are at higher risk of death as compared to patients with SMAVR.

To illustrate the data in more detail, Kaplan-Meier curves were plotted and the number of patients at risk, the number of censored patients and the number of events is presented for each year up to the 10-year follow up. Furthermore, survival probabilities (and corresponding 95% confidence intervals) were estimated using the Kaplan-Meier method.
Follow-up times were calculated using the reverse Kaplan-Meier survival curve.

Additional to the original model described above, the following sensitivity analyses were performed:

First, the model was repeated additionally accounting for the interaction between age and heart valve type as well as sex and heart valve type.

Second, the models with and without interaction terms were calculated for the subgroup of patients aged 50 - 60 years.

Third, due to potential unbalanced cohorts we performed propensity score matching (PSM) between the two groups (SMAVR and SBAVR). The balance between heart valve groups before and after propensity score matching is presented as standardized mean differences (SMD).
The propensity score was estimated with logistic regression based on age, sex and the following co-morbidities present 1 year before index surgery: Myocaridal Infarction, Embolic Stroke or ICH, Diabetes mellitus, Adiposity, Hyperlipidemia, Hyperuricemia/gout, Valvular, rhythmological and other CMPs, Ischemic CMP, Atherosclerosis, Pulmonary diseases, Kidney diseases and Malignant diseases.
The matching was performed for a 1:1 ratio using a nearest-neighbor matching algorithm with a caliper width of 0.01 standard deviations for the logit of the propensity score. Matching was performed for patients aged 50-65 as well as for the subgroup of patients aged 50 – 60, separately.
After propensity score matching, the associations between heart valve type and the time to death was evaluated using a mixed effect cox regression model using the matching ID as random factor. This model was accounting for heart valve type, age, sex and the co-morbidities Myocaridal Infarction, Embolic Stroke or ICH, Diabetes Mellitus, Adiposity, Hyperlipidemia, Hyperuricemia/Gout, Valvular-, rhythmological or other CMPs, Ischemic CMP, Artherosclerosis, Pulmonary diseases, Kidney diseases and Malignant diseases. The model was again repeated accounting for the interaction between age and heart valve type as well as sex and heart valve type. Furthermore, the models (with and without interaction terms) were calculated separately for the matched patients aged 50 - 65 as well as for the subgroup of patients aged 50 – 60.

3.2. Analyses of secondary outcomes

The combined secondary outcomes "MACE" as well as "death or reoperation" were analyzed similar to the primary endpoint “all-cause death”.

The time until the remaining events (heart failure, myocardial infarction, embolic stroke or ICH, reoperation, bleeding other than embolic stroke or ICH), were assessed using competing risk regression including death as competing event (using Fine and Grey method). Cumulative incidence functions were plotted and results of competing risk regression models are presented as sub-distribution hazard ratios and corresponding 95% confidence intervals as well as p-values. The number of patients at risk, the number of censored patients, the number of events and event probabilities (with corresponding 95% confidence intervals) are presented for each year up to the 10-year follow up. The models were accounting for all covariables as described for the original model. The models were repeated accounting for the interaction between age and heart valve type as well as sex and heart valve type. The models (with and without interaction terms) were calculated for the age group of patients aged 50 - 65 as well as for the subgroup of patients aged 50 - 60.

Furthermore, the models were repeated in the propensity score matched cohorts (see 3.1). To account for matching, associations were evaluated using competing risk regression including death as competing event and matching ID as clustering variable (using Fine and Grey method). Note that after propensity score matching the sample size and event rates for secondary endpoints were lower, i.e. not for all outcomes the full model including all co-morbidities could be calculated. Co-morbidities not included in the models are shown as “NA”. Cumulative incidence functions were plotted and results of competing risk regression models are presented as sub-distribution hazard ratios (with corresponding 95% confidence intervals) as well as p-values). The number of patients at risk, the number of censored patients, the number of events, event probabilities (with corresponding 95% confidence intervals) are presented for each year up to the 10-year follow up.

Further note, that in the analyses for “heart failure”, patients with a heart failure before Index-OP were excluded from the analyses to evaluate “newly diagnosed heart failures”.

3.3. Analyses of exploratory outcome

Due to the small number of events, time to all-cause death after reoperation was only evaluated in a descriptive way using Kaplan-Meier method.

The number of patients at risk, the number of censored patients and the number of events is presented for each year up to the 10-year follow up. Furthermore, survival probabilities (with corresponding 95% confidence intervals) were estimated using the Kaplan-Meier method.

3.4. Software

All analyses were performed using R, version 4.3.2. R-packages survival, survminer, coxme, crrSC were used for time-to event models and matchIt for propensity score matching.

4.) Additional Information on baseline characteristics

Medication	SMAVR (n = 1018)	SBAVR (n = 2743)	p-value
Therapies for hepatobiliary diseases	4 (0.39%)	8 (0.29%)	0.870
Intestinal anti-infectives	9 (0.88%)	36 (1.31%)	0.366
Intestinal anti-inflammatory drugs (steroids)	5 (0.49%)	8 (0.29%)	0.539
Insulins	37 (3.63%)	160 (5.83%)	0.009
Non-insulin antidiabetics (e.g., SLGT2i and GLP1-RA)	111 (10.9%)	421 (15.35%)	<0.001
Vitamin D and analogues	27 (2.65%)	124 (4.52%)	0.012
Calcium, potassium, magnesium salts	58 (5.7%)	218 (7.95%)	0.023
Vitamin K antagonists	76 (7.47%)	121 (4.41%)	<0.001
Heparins (UFH and LMWH)	160 (15.72%)	372 (13.56%)	0.103
Platelet aggregation inhibitors	110 (10.81%)	410 (14.95%)	0.001
Direct thrombin inhibitors and Fxa inhibitors (anticoagulants)	51 (5.01%)	129 (4.7%)	0.760
Iron substitution	16 (1.57%)	68 (2.48%)	0.121
Anti-anemic drugs (ESA, HIFi)	5 (0.49%)	37 (1.35%)	0.04
Glycosides	12 (1.18%)	30 (1.09%)	0.963
Antiarrhythmic drugs (excluding digitalis glycosides)	21 (2.06%)	83 (3.03%)	0.137
Adrenergic and dopaminergic stimulants (including PDE)	6 (0.59%)	15 (0.55%)	1
Vasodilators	58 (5.7%)	230 (8.38%)	0.007
Antihypertensives (excluding BB, MRA, loop diuretics, RAASi)	170 (16.7%)	553 (20.16%)	0.019
New therapeutics for PHA	0 (0%)	2 (0.07%)	0.948
Loop diuretics	67 (6.58%)	216 (7.87%)	0.205
MRA	85 (8.35%)	236 (8.6%)	0.856
Beta-blockers	296 (29.08%)	814 (29.68%)	0.751
RAAS inhibitors	446 (43.81%)	1240 (45.21%)	0.467
Fat-lowering agents (statins, fibrates, and others)	459 (45.09%)	1350 (49.22%)	0.027
Topical antibiotics	17 (1.67%)	57 (2.08%)	0.504
Dermal steroids	48 (4.72%)	181 (6.6%)	0.039
Parathyroid antagonists	5 (0.49%)	25 (0.91%)	0.28
Systemic antibiotics	436 (42.83%)	1245 (45.39%)	0.172
Systemic antiviral drugs	24 (2.36%)	63 (2.3%)	1
Chemotherapies, including immunotherapies	3 (0.29%)	30 (1.09%)	0.033
Immunosuppressants	30 (2.95%)	81 (2.95%)	1
NSAIDs	283 (27.8%)	855 (31.17%)	0.05
Gout medication	44 (4.32%)	149 (5.43%)	0.198
Biophosphonates	12 (1.18%)	49 (1.79%)	0.244
Non-NSAID analgesics, including opioids	132 (12.97%)	341 (12.43%)	0.701
Inhalants against COPD	174 (17.09%)	598 (21.8%)	0.002
Xanthines and leukotriene antagonists	15 (1.47%)	47 (1.71%)	0.712
Direct thrombin inhibitors and Fxa inhibitors (anticoagulants) or vitamin K antagonists	126 (12.38%)	244 (8.9%)	0.002

Table S6: Prescribed medications the year prior to the index surgery

5.) Primary Outcome: All-Cause Death

The following two tables give the number of patients at risk, number of events, number of censored patients as well as the estimated survival probability and corresponding 95% confidence intervals for each year up to the 10 years follow up for patients aged 50 - 65 and the subgroup of patients aged 50 - 60 before (Table S7) and after (Table S8) propensity score matching.

Table S9 presents the results of the multivariable cox proportional hazard model (hazard ratios and corresponding 95% confidence intervals as well as p-values) for patients aged 50 - 65 and the subgroup of patients aged 50 - 60 before propensity score matching as well as the results of the multivariable mixed effects cox regression model (hazard ratios and corresponding 95% confidence intervals as well as p-values) for patients aged 50 - 65 and the subgroup of patients aged 50 - 60 after propensity score matching. From the performed models including interaction terms, only the interaction terms are presented separately.

Figure S2 presents Kaplan-Meier curves and 95% confidence intervals for all-cause death before (A,C) and after (B,D) PSM for all patients aged 50 – 65 years (A,B) and Patients aged 50 – 60 years (C,D).

		Patients aged 50 - 65					Patients aged 50 – 60				
Group	Year	N at risk	N events	N censored	Survival	95% CI	N at risk	N events	N censored	Survival	95% CI
SMAVR	0	1018	2	0	0.998	0.995 - 1	809	2	0	0.998	0.994 - 1
	1	990	26	0	0.972	0.963 - 0.983	793	14	0	0.980	0.971 - 0.990
	2	901	12	77	0.96	0.948 - 0.972	713	8	72	0.970	0.958 - 0.982
	3	818	14	69	0.945	0.931 - 0.959	639	10	64	0.956	0.941 - 0.970
	4	705	17	96	0.924	0.907 - 0.941	548	11	80	0.938	0.921 - 0.956
	5	585	16	104	0.902	0.882 - 0.922	449	9	90	0.922	0.902 - 0.942
	6	483	9	93	0.887	0.865 - 0.909	369	5	75	0.911	0.888 - 0.933
	7	386	8	89	0.871	0.847 - 0.895	296	3	70	0.903	0.879 - 0.927
	8	306	8	72	0.851	0.824 - 0.878	234	6	56	0.882	0.855 - 0.911
	9	205	9	92	0.821	0.788 - 0.854	153	8	73	0.845	0.808 - 0.883
	10	126	8	71	0.781	0.741 - 0.823	87	7	59	0.797	0.749 - 0.848
SBAVR	0	2743	5	0	0.998	0.997 - 1	1252	2	0	0.998	0.996 – 1
	1	2618	120	0	0.954	0.947 - 0.962	1205	45	0	0.962	0.952 - 0.973
	2	2359	38	221	0.94	0.931 - 0.949	1078	18	109	0.948	0.935 - 0.96
	3	2026	49	284	0.919	0.909 - 0.930	922	18	138	0.931	0.916 - 0.945
	4	1707	66	253	0.887	0.875 - 0.900	778	25	119	0.904	0.887 - 0.922
	5	1425	45	237	0.862	0.848 - 0.876	647	19	112	0.88	0.860 - 0.900
	6	1167	48	210	0.831	0.815 - 0.847	540	20	87	0.851	0.828 - 0.874
	7	936	48	183	0.793	0.775 - 0.812	431	23	86	0.812	0.785 - 0.839
	8	701	38	197	0.757	0.736 - 0.779	332	14	85	0.782	0.753 - 0.813
	9	489	35	177	0.713	0.689 - 0.738	242	14	76	0.744	0.710 - 0.780
	10	300	14	175	0.687	0.661 - 0.715	158	4	80	0.729	0.693 - 0.767

Table S7: Number of patients at risk, number of events, number of censored patients as well as the estimated survival probability and corresponding 95% confidence intervals for patients aged 50-65 and the subgroup of patients aged 50-60.

		PSM matched Patients aged 50 - 65					PSM matched Patients aged 50 – 60				
Group	Year	N at risk	N events	N censored	Survival	95% CI	N at risk	N events	N censored	Survival	95% CI
SMAVR	0	902	1	0	0.999	0.997 – 1	674	0	0	1	1 – 1
	1	877	24	0	0.972	0.962 - 0.983	662	12	0	0.982	0.972 - 0.992
	2	802	10	65	0.961	0.948 - 0.974	596	7	59	0.971	0.959 - 0.984
	3	727	13	62	0.945	0.930 - 0.96	533	9	54	0.956	0.940 - 0.972
	4	623	16	88	0.923	0.904 - 0.941	457	10	66	0.937	0.917 - 0.957
	5	523	15	85	0.899	0.878 - 0.921	377	7	73	0.922	0.900 - 0.944
	6	428	8	87	0.884	0.861 - 0.908	306	5	66	0.908	0.884 - 0.933
	7	343	7	78	0.868	0.842 - 0.894	248	2	56	0.902	0.876 - 0.929
	8	277	8	58	0.846	0.817 - 0.876	198	4	46	0.886	0.857 - 0.917
	9	185	8	84	0.816	0.782 - 0.852	132	7	59	0.847	0.807 - 0.888
	10	116	8	61	0.774	0.731 - 0.819	74	7	51	0.791	0.738 - 0.849
SBAVR	0	902	1	0	0.999	0.997 - 1.000	674	0	0	1	1 - 1
	1	875	26	0	0.97	0.959 - 0.981	653	21	0	0.969	0.956 - 0.982
	2	779	15	81	0.953	0.939 - 0.967	579	10	64	0.954	0.938 - 0.970
	3	669	11	99	0.938	0.923 - 0.955	497	9	73	0.938	0.919 - 0.957
	4	562	19	88	0.910	0.890 - 0.930	425	9	63	0.920	0.898 - 0.942
	5	472	12	78	0.888	0.866 - 0.912	355	9	61	0.898	0.873 - 0.924
	6	391	16	65	0.857	0.830 - 0.884	301	9	45	0.874	0.845 - 0.904
	7	313	15	63	0.821	0.790 - 0.853	235	12	54	0.835	0.801 - 0.871
	8	229	13	71	0.782	0.746 - 0.819	175	7	53	0.807	0.768 - 0.848
	9	163	13	53	0.730	0.688 - 0.775	133	8	34	0.765	0.719 - 0.813
	10	111	2	50	0.718	0.673 - 0.765	97	1	35	0.759	0.712 - 0.809

Table S8: Number of patients at risk, number of events, number of censored patients as well as the estimated survival probability for the propensity score matched cohorts of patients aged 50-65 and the subgroup of patients aged 50-60.

	Patients aged 50 – 65 years				Patients aged 50 – 60 years			
	All data		After PSM		All data		After PSM	
Variables	HR (95% CI)	P-value	HR (95% CI)	P-value	HR (95% CI)	P-value	HR (95% CI)	P-value
Original Model								
Heart valve (SMAVR)	1.352 (1.109 - 1.649)	0.003	1.400 (1.103 - 1.778)	0.006	1.601 (1.235 - 2.074)	<0.001	1.655 (1.213 - 2.258)	0.001
Age	1.036 (1.016 - 1.057)	<0.001	1.067 (1.036 - 1.099)	<0.001	1.054 (1.013 - 1.097)	0.009	1.072 (1.015 - 1.133)	0.013
Sex (M)	1.011 (0.849 - 1.205)	0.900	0.804 (0.606 - 1.068)	0.133	1.122 (0.862 - 1.459)	0.392	1.001 (0.695 - 1.441)	0.997
Heart failure (Yes)	1.643 (1.328 - 2.033)	<0.001	1.469 (1.022 - 2.111)	0.038	1.451 (1.017 - 2.07)	0.040	1.206 (0.670 - 2.170)	0.533
Myocardial infarction (Yes)	1.068 (0.718 - 1.587)	0.745	0.993 (0.496 - 1.988)	0.983	0.815 (0.395 - 1.682)	0.581	0.654 (0.154 - 2.778)	0.068
Embolic stroke or ICH (Yes)	1.699 (1.016 - 2.841)	0.043	2.909 (1.264 - 6.698)	0.012	1.709 (0.874 - 3.344)	0.117	3.219 (0.916 - 11.31)	0.074
Diabetes mellitus (Yes)	1.640 (1.35 - 1.992)	<0.001	1.407 (1.014 - 1.952)	0.041	1.703 (1.246 - 2.328)	0.001	1.761 (1.105 - 2.808)	0.017
Adiposity (Yes)	1.430 (1.123 - 1.821)	0.004	1.532 (1.037 - 2.263)	0.032	1.458 (1.01 - 2.105)	0.044	1.152 (0.634 - 2.093)	0.643
Hyperlipidemia (Yes)	0.674 (0.551 - 0.824)	<0.001	0.720 (0.527 - 0.985)	0.040	0.820 (0.609 - 1.102)	0.188	0.940 (0.621 - 1.425)	0.772
Hyperuricemia/gout (Yes)	1.271 (0.871 - 1.855)	0.213	1.320 (0.792 - 2.201)	0.287	1.471 (0.783 - 2.765)	0.23	1.109 (0.445 - 2.764)	0.825
Valvular, rhythmological, and other CMPs (Yes)	0.684 (0.552 - 0.848)	0.001	0.800 (0.580 - 1.104)	0.174	0.608 (0.449 - 0.825)	0.001	0.576 (0.390 - 0.851)	0.006
Ischemic CMP (Yes)	1.154 (0.977 - 1.362)	0.092	1.138 (0.877 - 1.477)	0.332	1.171 (0.912 - 1.504)	0.215	1.048 (0.747 - 1.472)	0.785
Atherosclerosis (Yes)	1.452 (1.063 - 1.984)	0.019	1.266 (0.700 - 2.288)	0.435	1.577 (0.926 - 2.686)	0.093	2.042 (1.001 - 4.165)	0.050
Pulmonary diseases (Yes)	2.014 (1.405 - 2.889)	<0.001	1.563 (0.819 - 2.983)	0.176	2.136 (1.202 - 3.795)	0.010	2.956 (1.361 - 6.418)	0.006
Kidney diseases (Yes)	2.330 (1.851 - 2.932)	<0.001	2.541 (1.765 - 3.659)	<0.001	2.371 (1.658 - 3.391)	<0.001	2.796 (1.650 - 4.738)	<0.001
Malignant diseases (Yes)	1.328 (0.928 - 1.9)	0.121	0.546 (0.199 - 1.494)	0.238	1.811 (1.079 - 3.039)	0.025	2.843 (0.967 - 8.356)	0.057
Interaction Terms								
Heart valve (SMAVR) × Age	0.965 (0.922 - 1.009)	0.118	0.979 (0.923 - 1.037)	0.466	1.017 (0.933 - 1.108)	0.703	1.044 (0.939 - 1.162)	0.427
Heart valve (SMAVR) × Sex (M)	1.105 (0.718 - 1.702)	0.649	0.974 (0.557 - 1.701)	0.926	1.164 (0.651 - 2.080)	0.609	0.881 (0.432 - 1.797)	0.727

Table S9: Hazard ratios (HRs) and corresponding 95% confidence intervals (CIs) from multivariable Cox regression models (additionally including a random effect for matching-ID for the matched population) accounting for all listed confounders for all-cause mortality in all patients and the subgroup of patients aged 50 - 60 years before and after propensity score matching (PSM).

Figure S2: Kaplan-Meier curves and 95% confidence intervals for all-cause death before (A,C) and after (B,D) PSM for all patients aged 50 – 65 years (A,B) and the subgroup of patients aged 50 – 60 years (C,D)

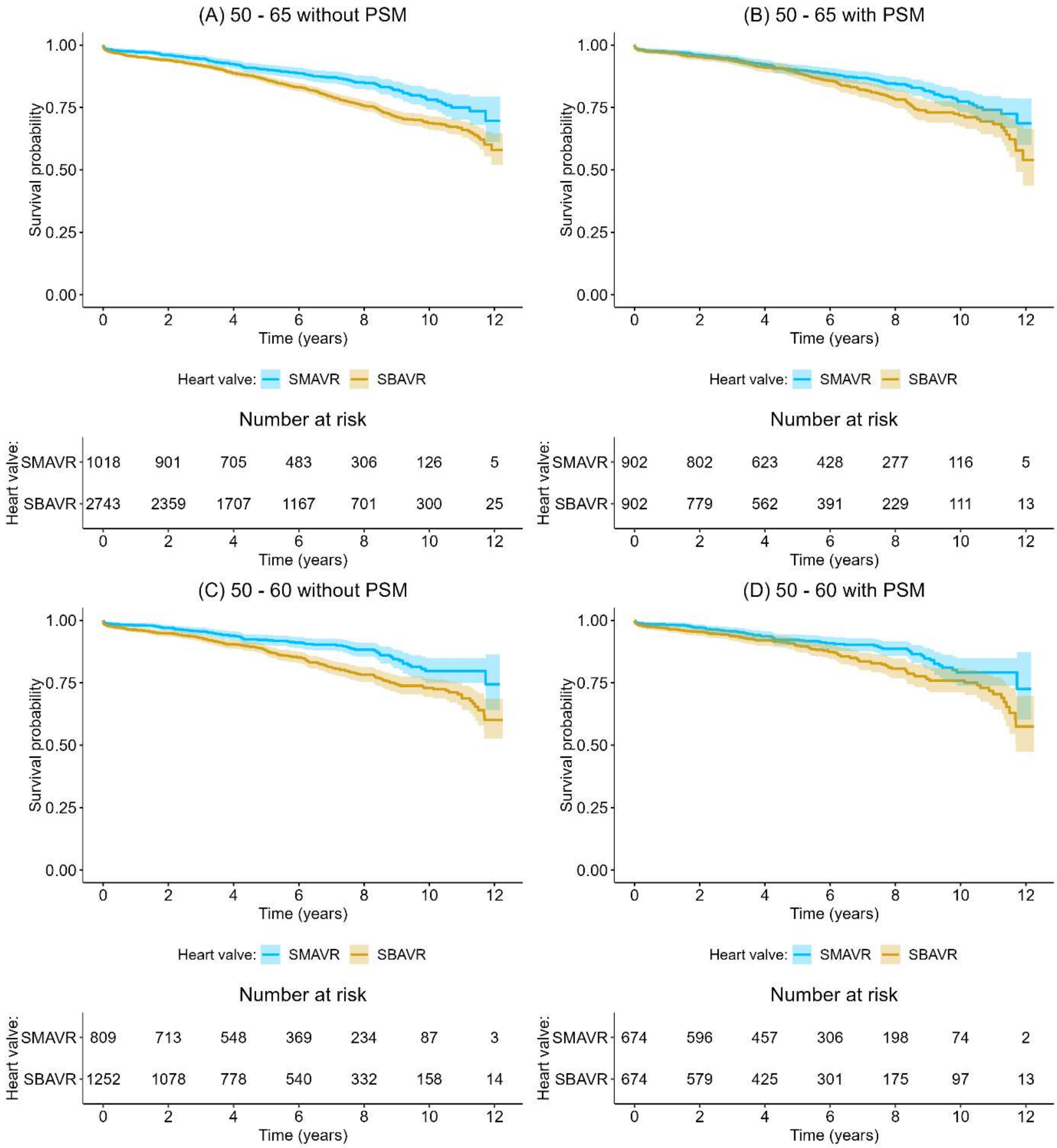

Secondary Outcomes

7.1. MACEs

The following two tables give the number of patients at risk, number of events, number of censored patients as well as the estimated MACE-free survival probability and corresponding 95% confidence intervals for each year up to the 10 years follow up for patients aged 50-65 and the subgroup of patients aged 50-60 before (Table S10) and after (Table S11) propensity score matching.

Table S12 presents the results of the multivariable cox proportional hazard model (hazard ratios and corresponding 95% confidence intervals as well as p-values) for patients aged 50-65 and the subgroup of patients aged 50-60 before propensity score matching as well as the results of the multivariable mixed effects cox regression model (hazard ratios and corresponding 95% confidence intervals as well as p-values) for patients aged 50-65 and the subgroup of patients aged 50-60 after propensity score matching. From the performed models including interaction terms, only the interaction terms are presented separately.

		Patients aged 50 – 65					Patients aged 50 - 60				
Group	Year	N at risk	N events	N censored	MACE-free	98% CI	N at risk	N events	N censored	MACE-free	98% CI
SMAVR	0	927	2	0	0.998	0.995 - 1	748	2	0	0.997	0.994 - 1
	1	767	84	74	0.906	0.887 - 0.925	615	62	69	0.913	0.893 - 0.933
	2	678	20	69	0.881	0.860 - 0.903	540	12	63	0.894	0.871 - 0.917
	3	573	18	87	0.856	0.833 - 0.880	452	13	75	0.871	0.846 - 0.897
	4	469	18	86	0.827	0.802 - 0.854	364	13	75	0.844	0.816 - 0.873
	5	377	13	79	0.803	0.775 - 0.832	289	8	67	0.824	0.793 - 0.855
	6	296	9	72	0.781	0.751 - 0.813	230	5	54	0.807	0.775 - 0.842
	7	224	10	62	0.752	0.718 - 0.788	175	4	51	0.792	0.757 - 0.829
	8	152	10	62	0.715	0.675 - 0.756	117	6	52	0.761	0.720 - 0.805
	9	92	6	54	0.684	0.639 - 0.731	67	5	45	0.724	0.675 - 0.777
	10	47	3	42	0.656	0.605 - 0.712	35	3	29	0.684	0.621 - 0.753
SBAVR	0	2448	2	0	0.999	0.998 - 1	1142	0	0	1	1 - 1
	1	1893	326	227	0.863	0.850 - 0.877	883	147	112	0.869	0.849 - 0.889
	2	1593	65	235	0.832	0.817 - 0.847	740	31	112	0.836	0.814 - 0.859
	3	1323	58	212	0.799	0.783 - 0.816	603	27	110	0.803	0.779 - 0.828
	4	1093	57	173	0.762	0.744 - 0.781	501	27	75	0.765	0.738 - 0.793
	5	863	47	183	0.727	0.707 - 0.747	403	15	83	0.741	0.712 - 0.770
	6	686	36	141	0.693	0.672 - 0.716	320	10	73	0.721	0.690 - 0.752
	7	502	37	147	0.651	0.626 - 0.676	252	13	55	0.689	0.655 - 0.723
	8	348	25	129	0.614	0.587 - 0.642	187	10	55	0.658	0.622 - 0.697
	9	203	18	127	0.575	0.545 - 0.607	113	9	65	0.619	0.578 - 0.664
	10	92	7	104	0.550	0.516 - 0.586	54	3	56	0.596	0.550 - 0.647

Table S10: Number of patients at risk, number of events, number of censored patients as well as the estimated MACE-free survival probability and corresponding 95% confidence intervals for patients aged 50-65 and the subgroup of patients aged 50-60.

		PSM matched Patients aged 50 - 65					PSM matched Patients aged 50 - 60				
Group	Year	N at risk	N events	N censored	MACE-free	98% CI	N at risk	N events	N censored	MACE-free	98% CI
SMAVR	0	828	1	0	0.999	0.996 - 1	635	0	0	1	1 - 1
	1	686	77	64	0.904	0.884 - 0.925	522	55	58	0.912	0.89 - 0.934
	2	606	18	62	0.880	0.857 - 0.903	456	11	55	0.891	0.867 - 0.917
	3	511	16	79	0.855	0.830 - 0.880	386	11	59	0.868	0.841 - 0.896
	4	421	17	73	0.825	0.797 - 0.853	308	12	66	0.84	0.809 - 0.871
	5	335	13	73	0.797	0.767 - 0.829	243	8	57	0.816	0.782 - 0.851
	6	263	9	63	0.773	0.740 - 0.808	193	5	45	0.797	0.760 - 0.835
	7	203	10	50	0.741	0.705 - 0.780	149	3	41	0.783	0.744 - 0.824
	8	139	9	55	0.705	0.664 - 0.749	100	5	44	0.754	0.709 - 0.801
	9	86	5	48	0.677	0.631 - 0.726	57	4	39	0.720	0.667 - 0.777
	10	43	3	40	0.648	0.594 - 0.706	28	3	26	0.672	0.603 - 0.750
SBAVR	0	824	1	0	0.999	0.996 - 1	627	0	0	1	1 – 1
	1	639	102	82	0.872	0.850 - 0.896	478	83	66	0.865	0.838 - 0.892
	2	535	21	83	0.842	0.816 - 0.868	405	17	56	0.832	0.802 - 0.863
	3	446	13	76	0.820	0.793 - 0.848	336	11	58	0.807	0.775 - 0.841
	4	374	19	53	0.782	0.752 - 0.814	285	10	41	0.782	0.747 - 0.818
	5	296	17	61	0.745	0.711 - 0.780	230	9	46	0.756	0.719 - 0.795
	6	233	11	52	0.714	0.678 - 0.753	180	6	44	0.735	0.696 - 0.777
	7	169	8	56	0.686	0.647 - 0.728	134	6	40	0.707	0.664 - 0.753
	8	126	9	34	0.645	0.601 - 0.693	102	6	26	0.672	0.623 - 0.725
	9	79	8	39	0.597	0.546 - 0.653	66	6	30	0.628	0.572 - 0.689
	10	42	3	34	0.569	0.512 - 0.632	35	2	29	0.601	0.538 - 0.672

Table S11: Number of patients at risk, number of events, number of censored patients as well as the estimated MACE-free survival probability and corresponding 95% confidence interval for the propensity score matched cohorts of patients aged 50-65 and the subgroup of patients aged 50-60.

	Patients aged 50 – 65 years				Patients aged 50 – 60 years			
	All data		After PSM		All data		After PSM	
Variables	HR (95% CI)	P-value	HR (95% CI)	P-value	HR (95% CI)	P-value	HR (95% CI)	P-value
Original Model								
Heart valve (SMAVR)	1.293 (1.093 - 1.531)	0.003	1.339 (1.097 - 1.636)	0.004	1.454 (1.180 - 1.792)	<0.001	1.475 (1.158 - 1.879)	0.002
Age	1.029 (1.012 - 1.047)	0.001	1.039 (1.014 - 1.065)	0.002	1.041 (1.007 - 1.075)	0.017	1.048 (1.004 - 1.094)	0.033
Sex (M)	0.987 (0.848 - 1.149)	0.866	0.848 (0.671 - 1.072)	0.169	1.110 (0.890 - 1.383)	0.354	1.012 (0.762 - 1.344)	0.935
Myocardial infarction (Yes)	1.585 (1.118 - 2.246)	0.01	1.637 (0.901 - 2.973)	0.106	1.129 (0.636 - 2.005)	0.679	1.415 (0.560 - 3.575)	0.463
Embolic stroke or ICH (Yes)	2.083 (1.371 - 3.162)	0.001	1.999 (0.926 - 4.318)	0.078	1.779 (0.990 - 3.195)	0.054	2.155 (0.650 - 7.147)	0.21
Diabetes mellitus (Yes)	1.755 (1.473 - 2.091)	<0.001	1.484 (1.110 - 1.984)	0.008	1.741 (1.326 - 2.287)	<0.001	1.762 (1.208 - 2.568)	0.003
Adiposity (Yes)	1.432 (1.145 - 1.791)	0.002	1.256 (0.871 - 1.810)	0.222	1.356 (0.972 - 1.891)	0.073	0.853 (0.504 - 1.445)	0.554
Hyperlipidemia (Yes)	0.788 (0.663 - 0.936)	0.007	0.911 (0.705 - 1.177)	0.474	0.926 (0.723 - 1.186)	0.542	1.037 (0.752 - 1.429)	0.826
Hyperuricemia/gout (Yes)	1.160 (0.793 - 1.696)	0.446	1.148 (0.680 - 1.936)	0.605	1.275 (0.689 - 2.360)	0.439	1.100 (0.457 - 2.645)	0.832
Valvular, rhythmological, and other CMPs (Yes)	0.767 (0.642 - 0.916)	0.003	0.824 (0.635 - 1.068)	0.144	0.695 (0.542 - 0.892)	0.004	0.743 (0.547 - 1.011)	0.058
Ischemic CMP (Yes)	1.022 (0.884 - 1.181)	0.77	1.117 (0.898 - 1.390)	0.321	1.139 (0.924 - 1.404)	0.222	1.002 (0.767 - 1.308)	0.99
Atherosclerosis (Yes)	1.507 (1.128 - 2.014)	0.006	1.419 (0.863 - 2.333)	0.167	1.312 (0.802 - 2.148)	0.280	1.337 (0.694 - 2.575)	0.386
Pulmonary diseases (Yes)	1.424 (0.990 - 2.047)	0.057	1.105 (0.601 - 2.031)	0.748	1.811 (1.085 - 3.023)	0.023	1.894 (0.963 - 3.725)	0.064
Kidney diseases (Yes)	2.041 (1.618 - 2.574)	<0.001	2.150 (1.510 - 3.060)	<0.001	2.201 (1.574 - 3.079)	<0.001	2.369 (1.498 - 3.746)	<0.001
Malignant diseases (Yes)	0.923 (0.622 - 1.370)	0.692	0.218 (0.054 - 0.881)	0.033	1.317 (0.752 - 2.306)	0.336	1.203 (0.373 - 3.882)	0.757
Interaction Terms								
Heart valve (SMAVR) × Age	0.966 (0.929 - 1.004)	0.076	0.969 (0.923 - 1.017)	0.201	0.984 (0.918 - 1.055)	0.646	1.005 (0.924 -1.092)	0.915
Heart valve (SMAVR) × Sex (M)	0.961 0.672 - 1.374)	0.828	0.938 (0.592 - 1.486)	0.785	0.927 (0.585 - 1.469)	0.746	0.751 (0.433 - 1.303)	0.309

Table S12: Hazard ratios (HRs) and corresponding 95% confidence intervals (CIs) from multivariable Cox regression accounting for all listed confounders for MACEs in all patients and the subgroup of patients aged 50 – 60 years before and after propensity score matching (PSM)

Figure S3: Kaplan-Meier curves and 95% confidence intervals for MACE free survival before (A,C) and after (B,D) PSM for all patients aged 50 – 65 years (A,B) and the subgroup of patients aged 50 – 60 years (C,D)

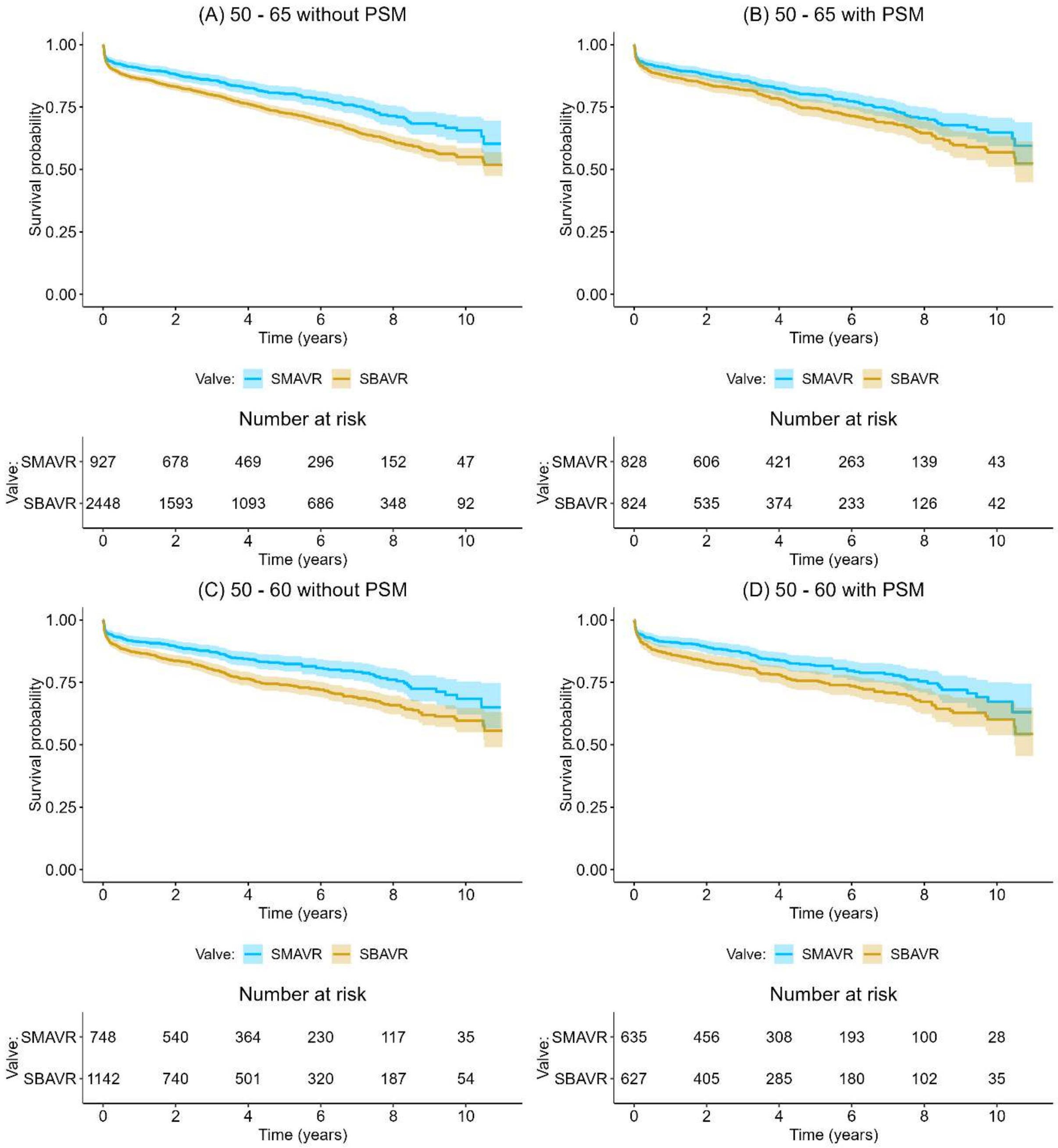

7.2. Death or reoperation

The following two tables give the number of patients at risk, number of events, number of censored patients as well as the estimated reoperation-free survival probability and corresponding 95% confidence intervals for each year up to the 10 years follow up for patients aged 50-65 and the subgroup of patients aged 50-60 before (Table S13) and after (Table S14) propensity score matching.

Table S15 presents the results of the multivariable cox proportional hazard model (hazard ratios and corresponding 95% confidence intervals as well as p-values) for patients aged 50-65 and the subgroup of patients aged 50-60 before propensity score matching as well as the results of the multivariable mixed effects cox regression model (hazard ratios and corresponding 95% confidence intervals as well as p-values) for patients aged 50-65 and the subgroup of patients aged 50-60 after propensity score matching. From the performed models including interaction terms, only the interaction terms are presented separately.

		Patients aged 50 - 65					Patients aged 50 - 60				
Group	Year	N at risk	N events	N censored	Re-OP free	95% CI	N at risk	N events	N censored	Re-OP free	95% CI
SMAVR	0	1018	2	0	0.998	0.995 - 1	809	2	0	0.998	0.994 - 1
	1	892	35	89	0.963	0.951 - 0.975	701	22	84	0.969	0.957 - 0.982
	2	805	10	77	0.952	0.938 - 0.965	624	7	70	0.959	0.945 - 0.973
	3	688	13	104	0.935	0.919 - 0.951	529	10	85	0.943	0.926 - 0.960
	4	571	12	105	0.918	0.900 - 0.936	433	7	89	0.929	0.910 - 0.949
	5	464	14	93	0.894	0.872 - 0.916	348	7	78	0.914	0.892 - 0.936
	6	370	7	87	0.878	0.854 - 0.903	279	4	65	0.901	0.877 - 0.927
	7	283	8	79	0.858	0.831 - 0.886	213	3	63	0.891	0.864 - 0.919
	8	192	8	83	0.830	0.798 - 0.864	141	6	66	0.862	0.828 - 0.898
	9	119	5	68	0.805	0.767 - 0.844	83	4	54	0.831	0.787 - 0.878
	10	60	4	55	0.771	0.724 - 0.822	41	4	38	0.781	0.719 - 0.848
SBAVR	0	2743	5	0	0.998	0.997 - 1	1252	2	0	0.998	0.996 - 1
	1	2312	155	271	0.940	0.931 - 0.949	1057	63	130	0.947	0.934 - 0.959
	2	1989	39	284	0.923	0.913 - 0.934	901	19	137	0.929	0.914 - 0.944
	3	1688	47	254	0.900	0.888 - 0.912	753	20	128	0.906	0.889 - 0.924
	4	1401	65	222	0.863	0.848 - 0.877	637	22	94	0.878	0.858 - 0.899
	5	1142	38	221	0.837	0.821 - 0.853	525	15	97	0.856	0.833 - 0.879
	6	910	46	186	0.800	0.782 - 0.819	413	19	93	0.822	0.796 - 0.849
	7	672	45	193	0.755	0.734 - 0.777	317	21	75	0.776	0.745 - 0.808
	8	454	39	179	0.705	0.680 - 0.731	226	12	79	0.743	0.709 - 0.780
	9	264	31	159	0.645	0.615 - 0.677	140	15	71	0.683	0.640 - 0.728
	10	122	11	131	0.610	0.575 - 0.647	68	3	69	0.665	0.619 - 0.714

Table S13: Number of patients at risk, number of events, number of censored patients as well as the estimated Reoperation-free survival probability and corresponding 95% confidence intervals for patients aged 50-65 and the subgroup of patients aged 50-60.

		PSM matched Patients aged 50 - 65					PSM matched Patients aged 50 - 60				
Group	Year	N at risk	N events	N censored	Re-OP free	95% CI	N at risk	N events	N censored	Re-OP free	95% CI
SMAVR	0	902	1	0	0.999	0.997 - 1	674	0	0	1	1 - 1
	1	793	33	75	0.961	0.949 - 0.974	587	18	69	0.973	0.960 - 0.985
	2	715	9	69	0.950	0.936 - 0.965	520	6	61	0.962	0.947 - 0.977
	3	608	12	95	0.933	0.916 - 0.95	445	9	66	0.944	0.926 - 0.963
	4	510	11	87	0.915	0.896 - 0.935	362	6	77	0.931	0.909 - 0.952
	5	411	13	86	0.890	0.867 - 0.914	292	5	65	0.917	0.894 - 0.942
	6	328	7	76	0.873	0.847 - 0.899	234	4	54	0.903	0.875 - 0.931
	7	257	8	63	0.850	0.821 - 0.881	180	3	51	0.891	0.861 - 0.922
	8	175	7	75	0.824	0.790 - 0.860	120	4	56	0.868	0.832 - 0.906
	9	110	4	61	0.801	0.762 - 0.843	71	4	45	0.831	0.783 - 0.883
	10	55	4	51	0.766	0.716 - 0.819	34	4	33	0.773	0.704 - 0.849
SBAVR	0	902	1	0	0.999	0.997 - 1	674	0	0	1	1 - 1
	1	764	39	98	0.954	0.941 - 0.968	563	33	78	0.949	0.933 - 0.966
	2	650	14	100	0.936	0.920 - 0.953	486	9	68	0.934	0.914 - 0.953
	3	549	12	89	0.918	0.898 - 0.937	407	10	69	0.913	0.890 - 0.936
	4	462	22	65	0.878	0.854 - 0.903	348	9	50	0.891	0.865 - 0.918
	5	378	11	73	0.855	0.828 - 0.883	288	9	51	0.866	0.836 - 0.897
	6	300	15	63	0.818	0.787 - 0.851	224	10	54	0.832	0.798 - 0.869
	7	215	13	72	0.778	0.742 - 0.816	164	11	49	0.786	0.744 - 0.830
	8	154	10	51	0.736	0.694 - 0.781	124	6	34	0.754	0.707 - 0.804
	9	96	13	45	0.661	0.609 - 0.719	82	9	33	0.691	0.634 - 0.753
	10	52	2	42	0.639	0.580 - 0.703	46	1	35	0.683	0.625 - 0.746

Table S14: Number of patients at risk, number of events, number of censored patients as well as the estimated Reoperation-free survival probability for the propensity score matched cohorts of patients aged 50-65 and the subgroup of patients aged 50-60.

	Patients aged 50 – 65 years				Patients aged 50 – 60 years			
	All data		After PSM		All data		After PSM	
Variables	HR (95% CI)	P-value	HR (95% CI)	P-value	HR (95% CI)	P-value	HR (95% CI)	P-value
Original Model								
Heart valve (SMAVR)	1.550 (1.26 - 1.908)	<0.001	1.566 (1.223 - 2.005)	<0.001	1.732 (1.325 - 2.264)	<0.001	1.885 (1.373 - 2.587)	<0.001
Age	1.034 (1.014 - 1.054)	0.001	1.056 (1.024 - 1.088)	0.001	1.049 (1.007 - 1.092)	0.022	1.069 (1.013 - 1.128)	0.015
Sex (M)	1.037 (0.870 - 1.237)	0.682	0.885 (0.665 - 1.178)	0.402	1.156 (0.886 - 1.507)	0.286	1.013 (0.709 - 1.448)	0.943
Heart failure (Yes)	1.583 (1.271 - 1.971)	<0.001	1.562 (1.082 - 2.256)	0.017	1.548 (1.082 - 2.216)	0.017	1.327 (0.753 - 2.337)	0.328
Myocardial infarction (Yes)	1.092 (0.729 - 1.636)	0.671	1.128 (0.561 - 2.265)	0.736	0.997 (0.500 - 1.985)	0.992	1.070 (0.331 - 3.456)	0.910
Embolic stroke or ICH (Yes)	1.938 (1.194 - 3.145)	0.007	1.806 (0.657 - 4.965)	0.252	1.449 (0.712 - 2.948)	0.306	1.713 (0.414 - 7.089)	0.458
Diabetes mellitus (Yes)	1.593 (1.305 - 1.945)	<0.001	1.479 (1.052 - 2.078)	0.024	1.570 (1.135 - 2.171)	0.006	1.715 (1.086 - 2.708)	0.021
Adiposity (Yes)	1.277 (0.989 - 1.648)	0.061	1.391 (0.915 - 2.115)	0.122	1.234 (0.832 - 1.83)	0.296	0.709 (0.358 - 1.406)	0.325
Hyperlipidemia (Yes)	0.674 (0.548 - 0.828)	<0.001	0.693 (0.498 - 0.963)	0.029	0.798 (0.587 - 1.084)	0.149	0.979 (0.653 - 1.467)	0.918
Hyperuricemia/gout (Yes)	1.071 (0.705 - 1.629)	0.748	1.068 (0.593 - 1.926)	0.826	1.282 (0.645 - 2.549)	0.478	1.119 (0.436 - 2.871)	0.816
Valvular, rhythmological, and other CMPs (Yes)	0.708 (0.571 - 0.878)	0.002	0.831 (0.599 - 1.154)	0.269	0.654 (0.481 - 0.889)	0.007	0.648 (0.443 - 0.949)	0.026
Ischemic CMP (Yes)	1.098 (0.927 - 1.300)	0.279	1.067 (0.815 - 1.398)	0.635	1.131 (0.876 - 1.461)	0.344	1.019 (0.731 - 1.422)	0.910
Atherosclerosis (Yes)	1.443 (1.045 - 1.992)	0.026	1.422 (0.785 - 2.577)	0.245	1.706 (1.000 - 2.911)	0.050	2.456 (1.273 - 4.738)	0.007
Pulmonary diseases (Yes)	1.774 (1.183 - 2.661)	0.006	0.686 (0.253 - 1.859)	0.459	1.938 (1.042 - 3.602)	0.037	2.161 (0.931 - 5.017)	0.073
Kidney diseases (Yes)	2.172 (1.707 - 2.763)	<0.001	2.241 (1.523 - 3.298)	<0.001	1.994 (1.356 - 2.933)	<0.001	2.090 (1.197 - 3.651)	0.010
Malignant diseases (Yes)	1.210 (0.832 - 1.758)	0.319	0.612 (0.224 - 1.669)	0.337	1.683 (0.986 - 2.872)	0.056	3.111 (1.119 - 8.648)	0.030
Interaction Terms								
Heart valve (SMAVR) × Age	0.970 (0.925 - 1.017)	0.204	0.985 (0.927 - 1.045)	0.610	0.993 (0.908 - 1.085)	0.869	0.997 (0.893 - 1.112)	0.956
Heart valve (SMAVR) × Sex (M)	1.286 (0.811 - 2.038)	0.285	1.299 (0.734 - 2.301)	0.369	1.309 (0.714 - 2.399)	0.384	1.134 (0.546 - 2.354)	0.737

Supplementary Table S15: Hazard ratios (HRs) and corresponding 95% confidence intervals (CIs) from multivariable Cox regression accounting for all listed confounders for death or reoperation in all patients and the subgroup of patients aged 50-60 years before and after propensity score matching (PSM)

Figure S4: Kaplan-Meier curves and 95% confidence intervals reoperation free survival before (A,C) and after (B,D) PSM for all patients aged 50 – 65 years (A,B) and the subgroup of patients aged 50 – 60 years (C,D)

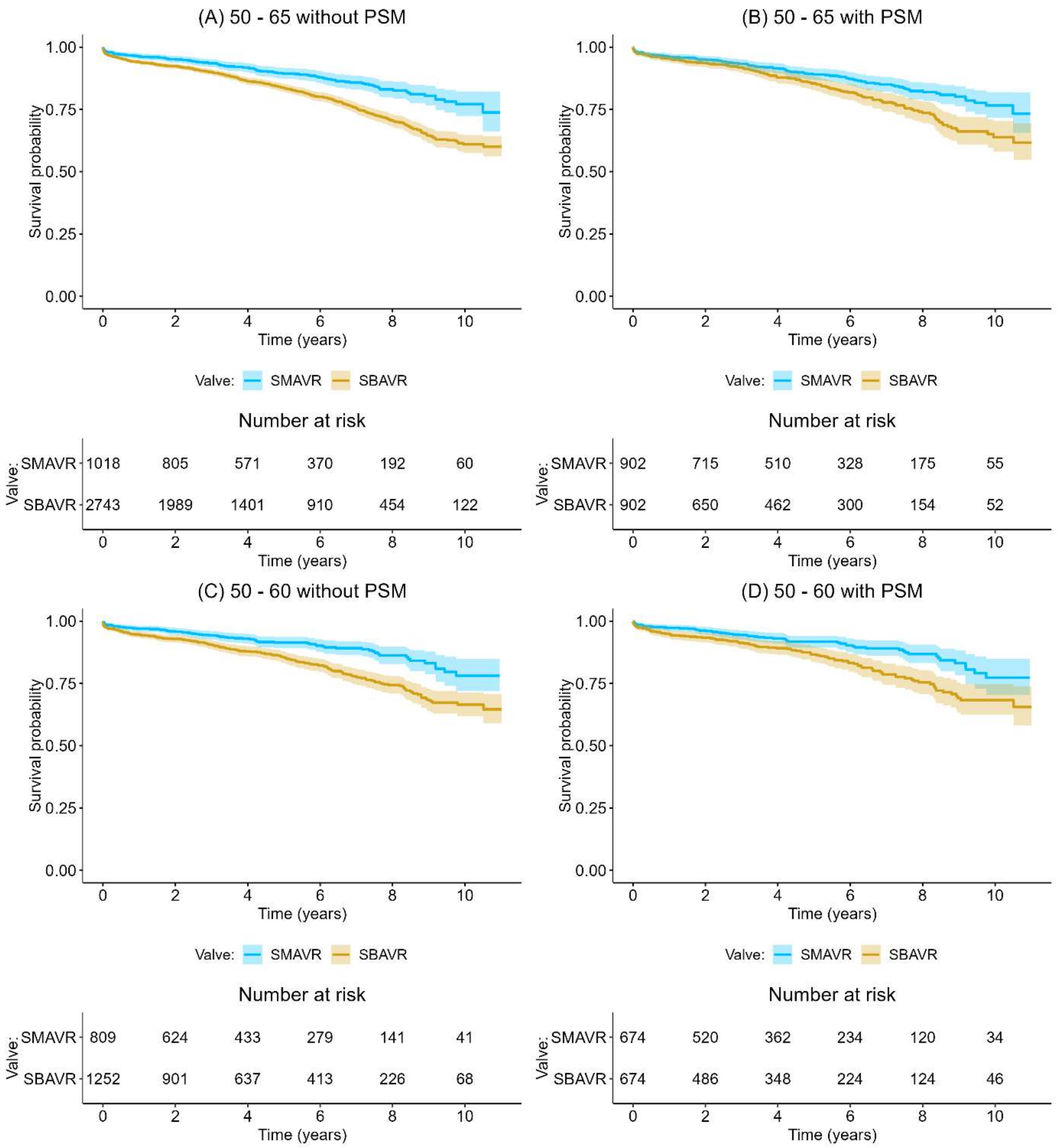

7.3. Reoperation

The following two tables give the number of patients at risk, number of events, number of censored patients as well as the estimated reoperation probability and corresponding 95% confidence intervals for each year up to the 10 years follow up for patients aged 50-65 and the subgroup of patients aged 50-60 before (Table S16) and after (Table S17) propensity score matching.

Table S18 presents the results of the multivariable competing risk models (sub-hazard ratios and corresponding 95% confidence intervals as well as p-values) for patients aged 50-65 and the subgroup of patients aged 50-60 before propensity score matching as well as the results of the multivariable competing risk model with clustering variable matching ID (sub-hazard ratios and corresponding 95% confidence intervals as well as p-values) for patients aged 50-65 and the subgroup of patients aged 50-60 after propensity score matching. From the performed models including interaction terms, only the interaction terms are presented separately.

		Patients aged 50 - 65					Patients aged 50 – 60				
Group	Year	N at risk	N events	N censored	Re-OP	95% CI	N at risk	N events	N censored	Re-OP	95% CI
SMAVR	0	1018	0	0	0	NA - NA	809	0	0	0	NA - NA
	1	892	9	89	0.009	0.005 - 0.017	701	8	84	0.010	0.005 - 0.020
	2	806	10	166	0.010	0.005 - 0.018	625	8	154	0.010	0.005 - 0.020
	3	688	12	270	0.013	0.007 - 0.022	529	10	239	0.014	0.007 - 0.024
	4	571	12	375	0.013	0.007 - 0.022	433	10	328	0.014	0.007 - 0.024
	5	465	12	468	0.013	0.007 - 0.022	349	10	406	0.014	0.007 - 0.024
	6	370	13	555	0.015	0.008 - 0.025	279	11	471	0.017	0.009 - 0.030
	7	283	14	634	0.017	0.010 - 0.029	213	12	534	0.020	0.010 - 0.035
	8	192	16	717	0.025	0.013 - 0.041	142	14	600	0.030	0.015 - 0.053
	9	120	16	785	0.025	0.013 - 0.041	84	14	654	0.030	0.015 - 0.053
	10	61	16	840	0.025	0.013 - 0.041	42	14	692	0.030	0.015 - 0.053
SBAVR	0	2743	0	0	0	NA - NA	1252	0	0	0	NA - NA
	1	2313	9	89	0.014	0.010 - 0.019	1059	8	84	0.015	0.009 - 0.023
	2	1989	10	166	0.018	0.013 - 0.023	901	8	154	0.021	0.014 - 0.030
	3	1689	12	270	0.021	0.016 - 0.027	754	10	239	0.024	0.016 - 0.034
	4	1402	12	375	0.027	0.021 - 0.034	638	10	328	0.028	0.019 - 0.039
	5	1143	12	468	0.030	0.024 - 0.038	525	10	406	0.032	0.023 - 0.045
	6	911	13	555	0.034	0.027 - 0.043	414	11	471	0.036	0.025 - 0.049
	7	673	14	634	0.046	0.036 - 0.057	318	12	534	0.040	0.028 - 0.055
	8	455	16	717	0.057	0.045 - 0.071	226	14	600	0.048	0.034 - 0.067
	9	264	16	785	0.067	0.053 - 0.083	140	14	654	0.056	0.039 - 0.078
	10	122	16	840	0.070	0.055 - 0.087	68	14	692	0.056	0.039 - 0.078

Table S16: Number of patients at risk, number of events, number of censored patients as well as the estimated Reoperation probability and corresponding 95% confidence intervals for patients aged 50-65 and the subgroup of patients aged 50-60.

		PSM matched Patients aged 50 - 65					PSM matched Patients aged 50 - 60				
Group	Year	N at risk	N events	N censored	Re-OP	95% CI	N at risk	N events	N censored	Re-OP	95% CI
SMAVR	0	902	0	0	0	NA - NA	674	0	0	0	NA - NA
	1	793	9	75	0.010	0.005 - 0.019	587	6	69	0.009	0.004 - 0.019
	2	716	10	144	0.012	0.006 - 0.021	521	6	130	0.009	0.004 - 0.019
	3	609	12	239	0.014	0.008 - 0.024	446	8	196	0.013	0.006 - 0.025
	4	510	12	326	0.014	0.008 - 0.024	362	8	273	0.013	0.006 - 0.025
	5	412	12	412	0.014	0.008 - 0.024	293	8	338	0.013	0.006 - 0.025
	6	328	13	488	0.017	0.009 - 0.029	234	9	392	0.017	0.008 - 0.032
	7	258	14	551	0.020	0.011 - 0.033	181	10	443	0.021	0.010 - 0.038
	8	175	15	626	0.023	0.013 - 0.039	121	11	499	0.026	0.013 - 0.049
	9	110	15	687	0.023	0.013 - 0.039	72	11	544	0.026	0.013 - 0.049
	10	56	15	738	0.023	0.013 - 0.039	35	11	577	0.026	0.013 - 0.049
SBAVR	0	902	0	0	0	NA - NA	674	0	0	0	NA - NA
	1	766	9	75	0.015	0.008 - 0.025	565	6	69	0.019	0.010 - 0.031
	2	651	10	144	0.020	0.012 - 0.031	487	6	130	0.024	0.014 - 0.038
	3	549	12	239	0.023	0.014 - 0.035	407	8	196	0.026	0.015 - 0.041
	4	463	12	326	0.034	0.023 - 0.050	349	8	273	0.033	0.021 - 0.051
	5	378	12	412	0.040	0.027 - 0.058	288	8	338	0.042	0.026 - 0.062
	6	301	13	488	0.043	0.029 - 0.060	225	9	392	0.048	0.031 - 0.071
	7	215	14	551	0.049	0.033 - 0.069	164	10	443	0.052	0.034 - 0.077
	8	155	15	626	0.057	0.039 - 0.081	124	11	499	0.058	0.037 - 0.084
	9	97	15	687	0.068	0.045 - 0.098	82	11	544	0.071	0.045 - 0.105
	10	52	15	738	0.068	0.045 - 0.098	46	11	577	0.071	0.045 - 0.105

Table S17: Number of patients at risk, number of events, number of censored patients as well as the estimated Reoperation probability for the propensity score matched cohorts of patients aged 50-65 and the subgroup of patients aged 50-60.

	Patients aged 50 – 65 years				Patients aged 50 – 60 years			
	All data		After PSM		All data		After PSM	
Variables	Sub-HR (95% CI)	P-value	Sub-HR (95% CI)	P-value	Sub-HR (95% CI)	P-value	Sub-HR (95% CI)	P-value
Original Model								
Heart valve (SMAVR)	2.338 (1.360 - 4.019)	0.002	2.451 (1.319 - 4.555)	0.005	2.015 (1.075 - 3.778)	0.029	2.579 (1.242 - 5.352)	0.011
Age	0.994 (0.950 - 1.041)	0.810	0.955 (0.89 - 1.025)	0.210	0.961 (0.88 - 1.049)	0.380	1.024 (0.912 - 1.15)	0.690
Sex (M)	1.152 (0.773 - 1.716)	0.490	1.616 (0.896 - 2.913)	0.110	1.398 (0.779 - 2.509)	0.260	1.508 (0.744 - 3.056)	0.250
Heart failure (Yes)	1.116 (0.612 - 2.033)	0.720	1.523 (0.600 - 3.864)	0.380	1.386 (0.569 - 3.372)	0.470	1.237 (0.359 - 4.26)	0.740
Myocardial infarction (Yes)	0.299 (0.040 - 2.204)	0.240	1.114 (0.142 - 8.733)	0.920	0.712 (0.09 - 5.635)	0.750	2.428 (0.272 - 21.71)	0.430
Embolic stroke or ICH (Yes)	2.249 (0.850 - 5.951)	0.100	NA	NA	NA	NA	NA	NA
Diabetes mellitus (Yes)	1.403 (0.866 - 2.272)	0.170	1.500 (0.640 - 3.512)	0.350	0.989 (0.419 - 2.336)	0.980	0.744 (0.209 - 2.651)	0.650
Adiposity (Yes)	0.836 (0.426 - 1.638)	0.600	0.692 (0.203 - 2.356)	0.560	0.406 (0.100 - 1.652)	0.210	NA	NA
Hyperlipidemia (Yes)	0.778 (0.478 - 1.266)	0.310	0.842 (0.400 - 1.77)	0.650	0.819 (0.385 - 1.742)	0.600	0.962 (0.413 - 2.238)	0.930
Hyperuricemia/gout (Yes)	0.332 (0.046 - 2.384)	0.270	NA	NA	NA	NA	NA	NA
Valvular, rhythmological, and other CMPs (Yes)	1.273 (0.744 - 2.178)	0.380	1.492 (0.660 - 3.370)	0.340	1.438 (0.675 - 3.062)	0.350	1.468 (0.605 - 3.560)	0.400
Ischemic CMP (Yes)	0.802 (0.537 - 1.200)	0.280	0.828 (0.456 - 1.504)	0.540	0.897 (0.487 - 1.65)	0.730	0.755 (0.368 - 1.550)	0.440
Atherosclerosis (Yes)	0.675 (0.212 - 2.151)	0.510	1.797 (0.420 - 7.692)	0.430	1.298 (0.293 - 5.743)	0.730	1.998 (0.398 - 10.043)	0.400
Pulmonary diseases (Yes)	1.480 (0.537 - 4.078)	0.450	NA	NA	1.597 (0.373 - 6.837)	0.530	1.186 (0.134 - 10.507)	0.880
Kidney diseases (Yes)	1.094 (0.525 - 2.280)	0.810	0.594 (0.132 - 2.663)	0.500	0.880 (0.254 - 3.054)	0.840	1.089 (0.255 - 4.657)	0.910
Malignant diseases (Yes)	0.487 (0.117 - 2.031)	0.320	1.004 (0.127 - 7.915)	1	1.267 (0.296 - 5.423)	0.750	NA	NA
Interaction Terms								
Heart valve (SMAVR) × Age	1.072 (0.947 - 1.213)	0.270	1.081 (0.929 - 1.259)	0.310	0.981 (0.793 - 1.213)	0.860	1.040 (0.795 - 1.362)	0.770
Heart valve (SMAVR) × Sex (M)	1.414 (0.423 - 4.720)	0.570	3.266 (0.781 - 13.651)	0.100	1.353 (0.355 - 5.158)	0.660	1.485 (0.315 - 7.006)	0.620

Table S18: Sub-Hazard ratios (Sub-HRs) and corresponding 95% confidence intervals (CIs) from multivariable competing risk regression models accounting for all listed confounders for reoperation in all patients and the subgroup of patients aged 50-60 years before and after propensity score matching (PSM)

Figure S5: Cumulative incidence curves for reoperation before (A,C) and after (B,D) PSM for all patients aged 50 – 65 years (A,B) and the subgroup of patients aged 50 – 60 years (C,D)

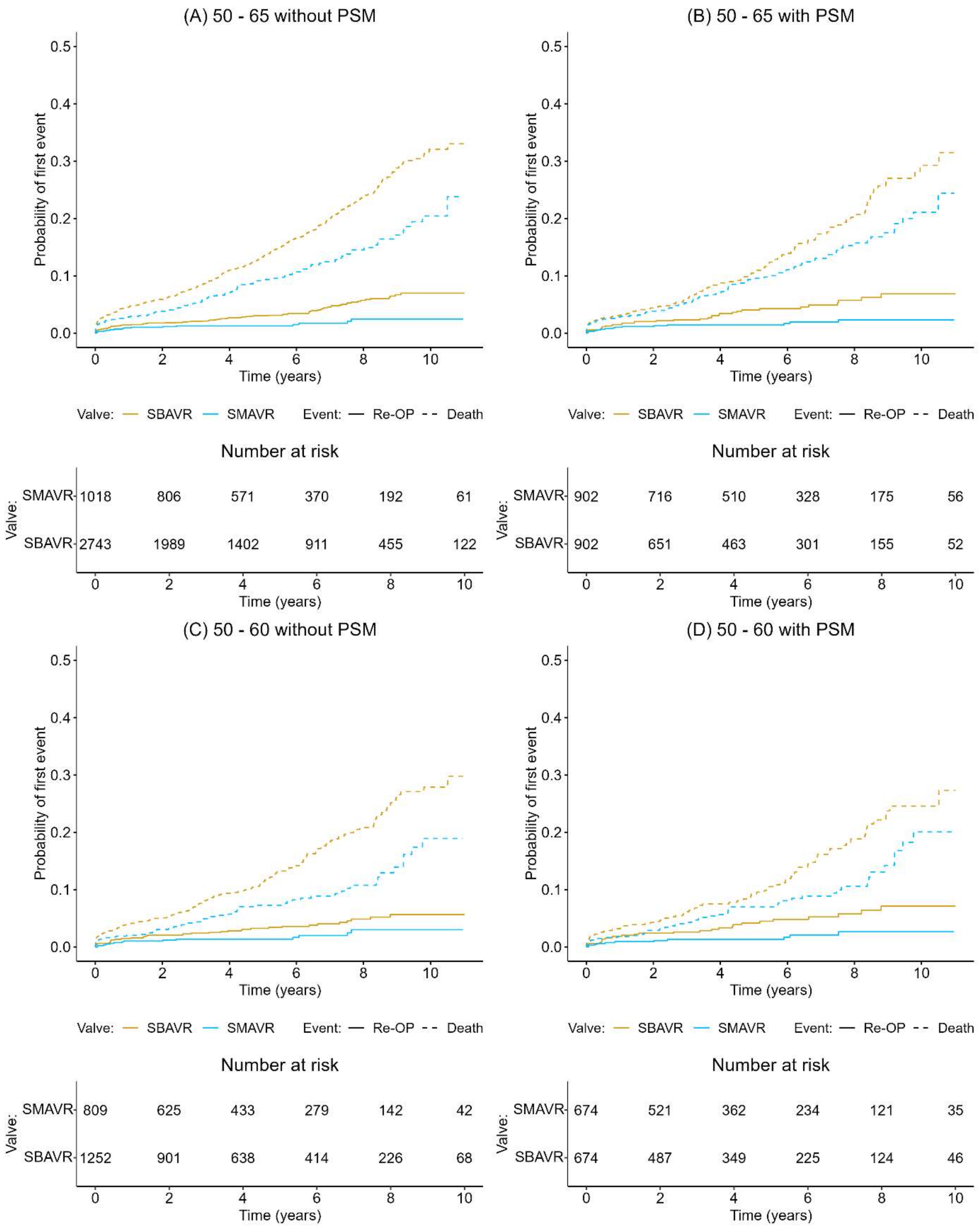

7.4. Heart failure

The following two tables give the number of patients at risk, number of events, number of censored patients as well as the estimated heart failure probability and corresponding 95% confidence intervals for each year up to the 10 years follow up for patients aged 50-65 and the subgroup of patients aged 50-60 before (Table S19) and after (Table S20) propensity score matching. Note that patients with a heart failure before Index-OP were excluded from these analyses.

Table S21 presents the results of the multivariable competing risk models (sub-hazard ratios and corresponding 95% confidence intervals as well as p-values) for patients aged 50-65 and the subgroup of patients aged 50-60 before propensity score matching as well as the results of the multivariable competing risk model with clustering variable matching ID (sub-hazard ratios and corresponding 95% confidence intervals as well as p-values) for patients aged 50-65 and the subgroup of patients aged 50-60 after propensity score matching. From the performed models including interaction terms, only the interaction terms are presented separately.

		Patients aged 50 - 65					Patients aged 50 - 60				
Group	Year	N at risk	N events	N censored	HF	95% CI	N at risk	N events	N censored	HF	95% CI
SMAVR	0	927	0	0	0	NA - NA	748	0	0	0	NA - NA
	1	838	47	20	0.051	0.038 - 0.066	680	37	18	0.049	0.036 - 0.067
	2	756	51	93	0.055	0.042 - 0.071	605	39	88	0.052	0.038 - 0.070
	3	665	60	169	0.067	0.052 - 0.084	526	45	156	0.062	0.046 - 0.081
	4	561	64	261	0.073	0.057 - 0.091	438	47	236	0.066	0.049 - 0.086
	5	464	71	343	0.084	0.066 - 0.105	359	52	304	0.077	0.058 - 0.099
	6	369	74	431	0.090	0.072 - 0.112	283	53	377	0.079	0.060 - 0.102
	7	295	80	496	0.106	0.084 - 0.131	231	56	424	0.090	0.068 - 0.115
	8	223	82	563	0.112	0.089 - 0.138	174	56	479	0.090	0.068 - 0.115
	9	149	82	633	0.112	0.089 - 0.138	109	56	540	0.090	0.068 - 0.115
	10	89	82	687	0.112	0.089 - 0.138	63	56	581	0.090	0.068 - 0.115
SBAVR	0	2448	0	0	0	NA - NA	1142	0	0	0	NA - NA
	1	2141	47	20	0.075	0.065 - 0.086	1012	37	18	0.067	0.053 - 0.082
	2	1845	51	93	0.086	0.075 - 0.098	871	39	88	0.074	0.060 - 0.091
	3	1557	60	169	0.098	0.086 - 0.110	733	45	156	0.089	0.073 - 0.107
	4	1279	64	261	0.103	0.091 - 0.116	598	47	236	0.094	0.077 - 0.112
	5	1074	71	343	0.116	0.103 - 0.130	504	52	304	0.107	0.089 - 0.127
	6	874	74	431	0.126	0.112 - 0.141	426	53	377	0.110	0.091 - 0.131
	7	685	80	496	0.131	0.116 - 0.146	327	56	424	0.114	0.095 - 0.136
	8	500	82	563	0.141	0.126 - 0.158	255	56	479	0.122	0.101 - 0.145
	9	336	82	633	0.15	0.133 - 0.169	175	56	540	0.130	0.107 - 0.155
	10	203	82	687	0.155	0.136 - 0.174	114	56	581	0.134	0.110 - 0.161

Table S19: Number of patients at risk, number of events, number of censored patients as well as the estimated heart failure probability and corresponding 95% confidence intervals for patients aged 50-65 and the subgroup of patients aged 50-60.

		PSM matched Patients aged 50 - 65					PSM matched Patients aged 50 - 60				
Group	Year	N at risk	N events	N censored	HF	95% CI	N at risk	N events	N censored	HF	95% CI
SMAVR	0	828	0	0	0	NA-NA	635	0	0	0	NA-NA
	1	751	42	15	0.051	0.037-0.067	579	32	15	0.05	0.035-0.069
	2	677	46	81	0.056	0.042-0.073	514	34	75	0.054	0.038-0.073
	3	595	55	150	0.069	0.052-0.088	445	40	134	0.065	0.047-0.087
	4	503	58	229	0.074	0.057-0.093	375	42	196	0.07	0.051-0.092
	5	415	65	303	0.087	0.067-0.108	304	46	257	0.08	0.059-0.104
	6	333	68	378	0.093	0.073-0.117	240	47	318	0.083	0.062-0.108
	7	266	74	437	0.111	0.087-0.137	197	49	358	0.091	0.068-0.119
	8	202	76	497	0.117	0.092-0.146	152	49	402	0.091	0.068-0.119
	9	137	76	558	0.117	0.092-0.146	96	49	454	0.091	0.068-0.119
	10	82	76	607	0.117	0.092-0.146	54	49	491	0.091	0.068-0.119
SBAVR	0	824	0	0	0	NA-NA	627	0	0	0	NA-NA
	1	730	42	15	0.067	0.051-0.085	558	32	15	0.065	0.048-0.087
	2	632	46	81	0.074	0.057-0.093	475	34	75	0.073	0.054-0.095
	3	533	55	150	0.083	0.065-0.103	408	40	134	0.083	0.062-0.106
	4	444	58	229	0.086	0.068-0.107	342	42	196	0.085	0.064-0.109
	5	370	65	303	0.106	0.085-0.13	287	46	257	0.103	0.079-0.13
	6	308	68	378	0.111	0.089-0.136	243	47	318	0.106	0.081-0.134
	7	241	74	437	0.116	0.093-0.143	185	49	358	0.113	0.087-0.143
	8	169	76	497	0.139	0.11-0.17	134	49	402	0.128	0.098-0.162
	9	122	76	558	0.144	0.114-0.177	101	49	454	0.142	0.107-0.181
	10	84	76	607	0.150	0.119-0.185	71	49	491	0.142	0.107-0.181

Table S20: Number of patients at risk, number of events, number of censored patients as well as the estimated heart failure probability for the propensity score matched cohorts of patients aged 50-65 and the subgroup of patients aged 50-60.

	Patients aged 50 – 65 years				Patients aged 50 – 60 years			
	All data		After PSM		All data		After PSM	
Variables	Sub-HR (95% CI)	P-value	Sub-HR (95% CI)	P-value	Sub-HR (95% CI)	P-value	Sub-HR (95% CI)	P-value
Original Model								
Heart valve (SMAVR)	1.181 (0.909 - 1.534)	0.210	1.286 (0.943 - 1.753)	0.11	1.379 (0.997 - 1.908)	0.052	1.457 (1.002 - 2.12)	0.049
Age	1.040 (1.012 - 1.069)	0.005	1.035 (0.998 - 1.074)	0.065	1.023 (0.975 - 1.074)	0.350	1.062 (0.997 - 1.132)	0.063
Sex (M)	0.981 (0.778 - 1.237)	0.870	0.867 (0.61 - 1.231)	0.420	1.082 (0.764 - 1.531)	0.660	0.969 (0.627 - 1.496)	0.890
Heart failure (Yes)	NA	NA	NA	NA	NA	NA	NA	NA
Myocardial infarction (Yes)	1.144 (0.696 - 1.882)	0.600	1.116 (0.477 - 2.613)	0.80	0.896 (0.409 - 1.966)	0.780	1.789 (0.678 - 4.721)	0.240
Embolic stroke or ICH (Yes)	2.548 (1.456 - 4.459)	0.001	2.596 (1.047 - 6.441)	0.040	2.142 (0.975 - 4.706)	0.058	1.392 (0.220 - 8.792)	0.730
Diabetes mellitus (Yes)	1.907 (1.483 - 2.452)	<0.001	1.762 (1.168 - 2.657)	0.007	1.886 (1.263 - 2.816)	0.002	1.951 (1.171 - 3.251)	0.010
Adiposity (Yes)	1.506 (1.107 - 2.047)	0.009	1.189 (0.706 - 2.001)	0.510	1.250 (0.762 - 2.05)	0.380	0.629 (0.271 - 1.462)	0.280
Hyperlipidemia (Yes)	0.734 (0.567 - 0.950)	0.019	0.831 (0.572 - 1.205)	0.330	1.031 (0.714 - 1.489)	0.870	1.025 (0.646 - 1.627)	0.910
Hyperuricemia/gout (Yes)	0.956 (0.508 - 1.797)	0.89	0.862 (0.347 - 2.141)	0.750	1.960 (0.914 - 4.206)	0.084	1.679 (0.581 - 4.853)	0.340
Valvular, rhythmological, and other CMPs (Yes)	0.780 (0.592 - 1.028)	0.078	0.735 (0.492 - 1.097)	0.130	0.696 (0.471 - 1.03)	0.070	0.665 (0.421 - 1.049)	0.079
Ischemic CMP (Yes)	1.086 (0.873 - 1.349)	0.460	1.280 (0.915 - 1.790)	0.150	1.235 (0.899 - 1.696)	0.190	1.075 (0.718 - 1.608)	0.730
Atherosclerosis (Yes)	1.630 (1.081 - 2.458)	0.020	1.775 (0.914 - 3.448)	0.090	1.793 (0.938 - 3.431)	0.078	1.831 (0.839 - 3.993)	0.130
Pulmonary diseases (Yes)	0.991 (0.548 - 1.794)	0.980	1.022 (0.405 - 2.578)	0.960	0.774 (0.275 - 2.178)	0.630	0.906 (0.244 - 3.372)	0.880
Kidney diseases (Yes)	1.997 (1.448 - 2.755)	<0.001	2.060 (1.257 - 3.376)	0.004	2.088 (1.301 - 3.351)	0.002	2.533 (1.406 - 4.562)	0.002
Malignant diseases (Yes)	1.058 (0.587 - 1.907)	0.850	NA	NA	1.612 (0.712 - 3.651)	0.250	NA	NA
Interaction Terms								
Heart valve (SMAVR) × Age	0.980 (0.923 - 1.040)	0.500	0.969 (0.9 - 1.043)	0.400	0.995 (0.899 - 1.102)	0.930	1.035 (0.910 - 1.177)	0.600
Heart valve (SMAVR) × Sex (M)	0.950 (0.551 - 1.637)	0.850	0.839 (0.408 - 1.723)	0.630	0.681 (0.335 - 1.384)	0.290	0.517 (0.218 - 1.226)	0.130

Table S21: Sub-Hazard ratios (Sub-HRs) and corresponding 95% confidence intervals (CIs) from multivariable competing risk regression models accounting for all listed confounders for heart failure in all patients and the subgroup of patients aged 50-60 years before and after propensity score matching (PSM)

Figure S6: Cumulative incidence curves for heart failure before (A,C) and after (B,D) PSM for all patients aged 50 – 65 years (A,B) and the subgroup of patients aged 50 – 60 years (C,D)

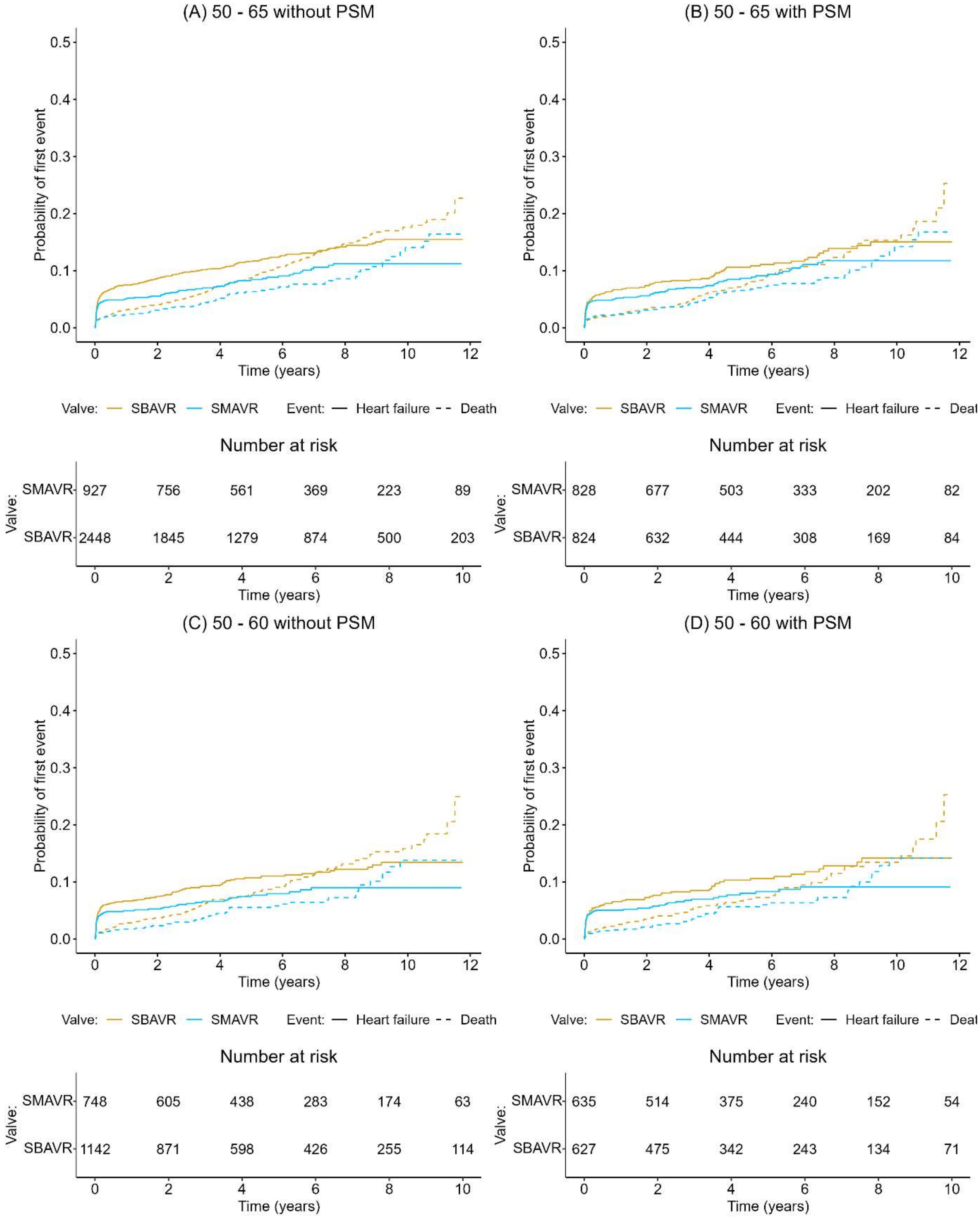

7.5. Myocardial infarction

The following two tables give the number of patients at risk, number of events, number of censored patients as well as the estimated myocardial infarction probability and corresponding 95% confidence intervals for each year up to the 10 years follow up for patients aged 50-65 and the subgroup of patients aged 50-60 before (Table S22) and after (Table S23) propensity score matching.

Table S24 presents the results of the multivariable competing risk models (sub-hazard ratios and corresponding 95% confidence intervals as well as p-values) for patients aged 50-65 and the subgroup of patients aged 50-60 before propensity score matching as well as the results of the multivariable competing risk model with clustering variable matching ID (sub-hazard ratios and corresponding 95% confidence intervals as well as p-values) for patients aged 50-65 and the subgroup of patients aged 50-60 after propensity score matching. From the performed models including interaction terms, only the interaction terms are presented separately.

		Patients aged 50 - 65					Patients aged 50 - 60				
Group	Year	N at risk	N events	N censored	MI	95% CI	N at risk	N events	N censored	MI	95% CI
SMAVR	0	1018	0	0	0	NA - NA	809	0	0	0	NA - NA
	1	962	5	23	0.005	0.002 - 0.011	768	4	21	0.005	0.002 - 0.012
	2	866	7	108	0.007	0.003 - 0.014	679	6	103	0.008	0.003 - 0.016
	3	767	8	192	0.008	0.004 - 0.016	595	6	177	0.008	0.003 - 0.016
	4	649	9	295	0.010	0.005 - 0.017	500	7	264	0.009	0.004 - 0.019
	5	536	10	393	0.011	0.006 - 0.020	409	8	345	0.012	0.005 - 0.022
	6	433	10	490	0.011	0.006 - 0.020	329	8	423	0.012	0.005 - 0.022
	7	349	10	566	0.011	0.006 - 0.020	269	8	479	0.012	0.005 - 0.022
	8	260	13	645	0.019	0.010 - 0.033	197	8	545	0.012	0.005 - 0.022
	9	171	14	727	0.023	0.012 - 0.039	125	8	612	0.012	0.005 - 0.022
	10	100	15	789	0.029	0.015 - 0.050	70	8	660	0.012	0.005 - 0.022
SBAVR	0	2743	0	0	0	NA - NA	1252	0	0	0	NA - NA
	1	2543	5	23	0.008	0.005 - 0.012	1168	4	21	0.007	0.004 - 0.013
	2	2210	7	108	0.009	0.006 - 0.013	1011	6	103	0.007	0.004 - 0.013
	3	1886	8	192	0.010	0.007 - 0.015	862	6	177	0.007	0.004 - 0.013
	4	1549	9	295	0.013	0.009 - 0.018	707	7	264	0.009	0.005 - 0.016
	5	1314	10	393	0.016	0.011 - 0.021	602	8	345	0.011	0.006 - 0.019
	6	1075	10	490	0.021	0.015 - 0.028	505	8	423	0.016	0.009 - 0.026
	7	827	10	566	0.027	0.020 - 0.035	383	8	479	0.021	0.013 - 0.034
	8	610	13	645	0.028	0.021 - 0.036	297	8	545	0.021	0.013 - 0.034
	9	403	14	727	0.031	0.023 - 0.040	197	8	612	0.027	0.016 - 0.043
	10	235	15	789	0.033	0.024 - 0.043	126	8	660	0.027	0.016 - 0.043

Table S22: Number of patients at risk, number of events, number of censored patients as well as the estimated myocardial infarction probability and corresponding 95% confidence intervals for patients aged 50-65 and the subgroup of patients aged 50-60.

		PSM matched Patients aged 50 - 65					PSM matched Patients aged 50 - 60				
Group	Year	N at risk	N events	N censored	MI	95% CI	N at risk	N events	N censored	MI	95% CI
SMAVR	0	902	0	0	0	NA - NA	674	0	0	0	NA - NA
	1	855	5	17	0.006	0.002 - 0.012	642	4	17	0.006	0.002 - 0.014
	2	770	7	92	0.008	0.004 - 0.016	566	6	86	0.009	0.004 - 0.019
	3	682	8	168	0.009	0.004 - 0.018	493	6	150	0.009	0.004 - 0.019
	4	578	9	257	0.011	0.005 - 0.02	419	7	217	0.011	0.005 - 0.023
	5	477	10	343	0.013	0.006 - 0.022	342	7	287	0.011	0.005 - 0.023
	6	388	10	427	0.013	0.006 - 0.022	276	7	351	0.011	0.005 - 0.023
	7	312	10	495	0.013	0.006 - 0.022	227	7	397	0.011	0.005 - 0.023
	8	234	13	563	0.021	0.011 - 0.037	170	7	451	0.011	0.005 - 0.023
	9	156	14	635	0.025	0.013 - 0.044	109	7	507	0.011	0.005 - 0.023
	10	93	15	689	0.031	0.016 - 0.055	61	7	548	0.011	0.005 - 0.023
SBAVR	0	902	0	0	0	NA - NA	674	0	0	0	NA - NA
	1	842	5	17	0.012	0.007 - 0.021	631	4	17	0.007	0.003 - 0.017
	2	726	7	92	0.013	0.007 - 0.023	541	6	86	0.007	0.003 - 0.017
	3	617	8	168	0.013	0.007 - 0.023	466	6	150	0.007	0.003 - 0.017
	4	511	9	257	0.023	0.014 - 0.036	388	7	217	0.012	0.005 - 0.023
	5	434	10	343	0.025	0.015 - 0.038	332	7	287	0.012	0.005 - 0.023
	6	360	10	427	0.029	0.018 - 0.044	280	7	351	0.014	0.007 - 0.028
	7	277	10	495	0.035	0.022 - 0.051	210	7	397	0.022	0.011 - 0.040
	8	197	13	563	0.035	0.022 - 0.051	156	7	451	0.022	0.011 - 0.040
	9	136	14	635	0.039	0.024 - 0.059	114	7	507	0.028	0.013 - 0.052
	10	92	15	689	0.039	0.024 - 0.059	79	7	548	0.028	0.013 - 0.052

Table S23: Number of patients at risk, number of events, number of censored patients as well as the estimated myocardial infarction probability for the propensity score matched cohorts of patients aged 50-65 and the subgroup of patients aged 50-60.

	Patients aged 50 – 65 years				Patients aged 50 – 60 years			
	All data		After PSM		All data		After PSM	
Variables	Sub-HR (95% CI)	P-value	Sub-HR (95% CI)	P-value	Sub-HR (95% CI)	P-value	Sub-HR (95% CI)	P-value
Original Model								
Heart valve (SMAVR)	1.187 (0.654 - 2.156)	0.570	1.799 (0.933 - 3.468)	0.080	1.421 (0.627 - 3.223)	0.400	1.560 (0.625 - 3.897)	0.340
Age	1.036 (0.970 - 1.108)	0.290	1.048 (0.959 - 1.145)	0.300	0.996 (0.863 - 1.149)	0.960	1.021 (0.871 - 1.197)	0.800
Sex (M)	0.968 (0.563 - 1.664)	0.910	0.957 (0.486 - 1.884)	0.900	0.229 (0.054 - 0.976)	0.046	0.378 (0.092 - 1.559)	0.180
Heart failure (Yes)	0.951 (0.454 - 1.992)	0.890	1.391 (0.516 - 3.752)	0.510	1.431 (0.443 - 4.624)	0.550	0.652 (0.092 - 4.609)	0.670
Myocardial infarction (Yes)	6.306 (3.089 - 12.873)	<0.001	3.487 (0.897 - 13.553)	0.071	3.650 (0.984 - 13.539)	0.053	2.611 (0.343 - 19.897)	0.350
Embolic stroke or ICH (Yes)	NA	NA	NA	NA	NA	NA	NA	NA
Diabetes mellitus (Yes)	1.597 (0.920 - 2.773)	0.096	1.624 (0.693 - 3.808)	0.260	2.178 (0.847 - 5.600)	0.110	2.011 (0.527 - 7.674)	0.310
Adiposity (Yes)	1.806 (0.929 - 3.513)	0.081	1.681 (0.59 - 4.788)	0.330	1.743 (0.499 - 6.095)	0.380	2.179 (0.359 - 13.226)	0.400
Hyperlipidemia (Yes)	1.150 (0.672 - 1.967)	0.610	1.496 (0.696 - 3.218)	0.300	1.649 (0.674 - 4.030)	0.270	1.624 (0.557 - 4.740)	0.370
Hyperuricemia/gout (Yes)	1.806 (0.694 - 4.700)	0.230	2.040 (0.825 - 5.043)	0.120	0.932 (0.120 - 7.22)	0.950	1.991 (0.296 - 13.403)	0.480
Valvular, rhythmological, and other CMPs (Yes)	0.823 (0.415 - 1.634)	0.580	0.551 (0.245 - 1.240)	0.150	0.363 (0.141 - 0.932)	0.035	0.278 (0.097 - 0.791)	0.016
Ischemic CMP (Yes)	0.976 (0.567 - 1.681)	0.930	1.089 (0.570 - 2.082)	0.800	1.130 (0.508 - 2.516)	0.760	1.452 (0.584 - 3.612)	0.420
Atherosclerosis (Yes)	1.928 (0.849 - 4.379)	0.120	3.286 (1.258 - 8.585)	0.015	1.774 (0.446 - 7.05)	0.420	1.401 (0.179 - 10.935)	0.750
Pulmonary diseases (Yes)	0.404 (0.056 - 2.920)	0.370	0.765 (0.099 - 5.925)	0.800	1.477 (0.206 - 10.614)	0.700	NA	NA
Kidney diseases (Yes)	0.851 (0.345 - 2.103)	0.730	1.070 (0.359 - 3.189)	0.900	0.499 (0.064 - 3.879)	0.510	1.142 (0.178 - 7.341)	0.890
Malignant diseases (Yes)	0.389 (0.053 - 2.876)	0.360	NA	NA	1.316 (0.194 - 8.915)	0.780	NA	NA
Interaction Terms								
Heart valve (SMAVR) × Age	0.912 (0.789 - 1.055)	0.210	0.958 (0.808 - 1.136)	0.620	0.861 (0.661 - 1.12)	0.260	1.119 (0.157 - 7.963)	0.91
Heart valve (SMAVR) × Sex (M)	0.353 (0.111 - 1.123)	0.078	0.224 (0.047 - 1.069)	0.061	NA	NA	NA	NA

Table S24: Sub-Hazard ratios (Sub-HRs) and corresponding 95% confidence intervals (CIs) from multivariable competing risk regression models accounting for all listed confounders for myocardial infarction in all patients and the subgroup of patients aged 50 - 60 years before and after propensity score matching (PSM)

Figure S7: Cumulative incidence curves for myocardial infarction before (A,C) and after (B,D) PSM for all patients aged 50 – 65 years (A,B) and the subgroup of patients aged 50 – 60 years (C,D)

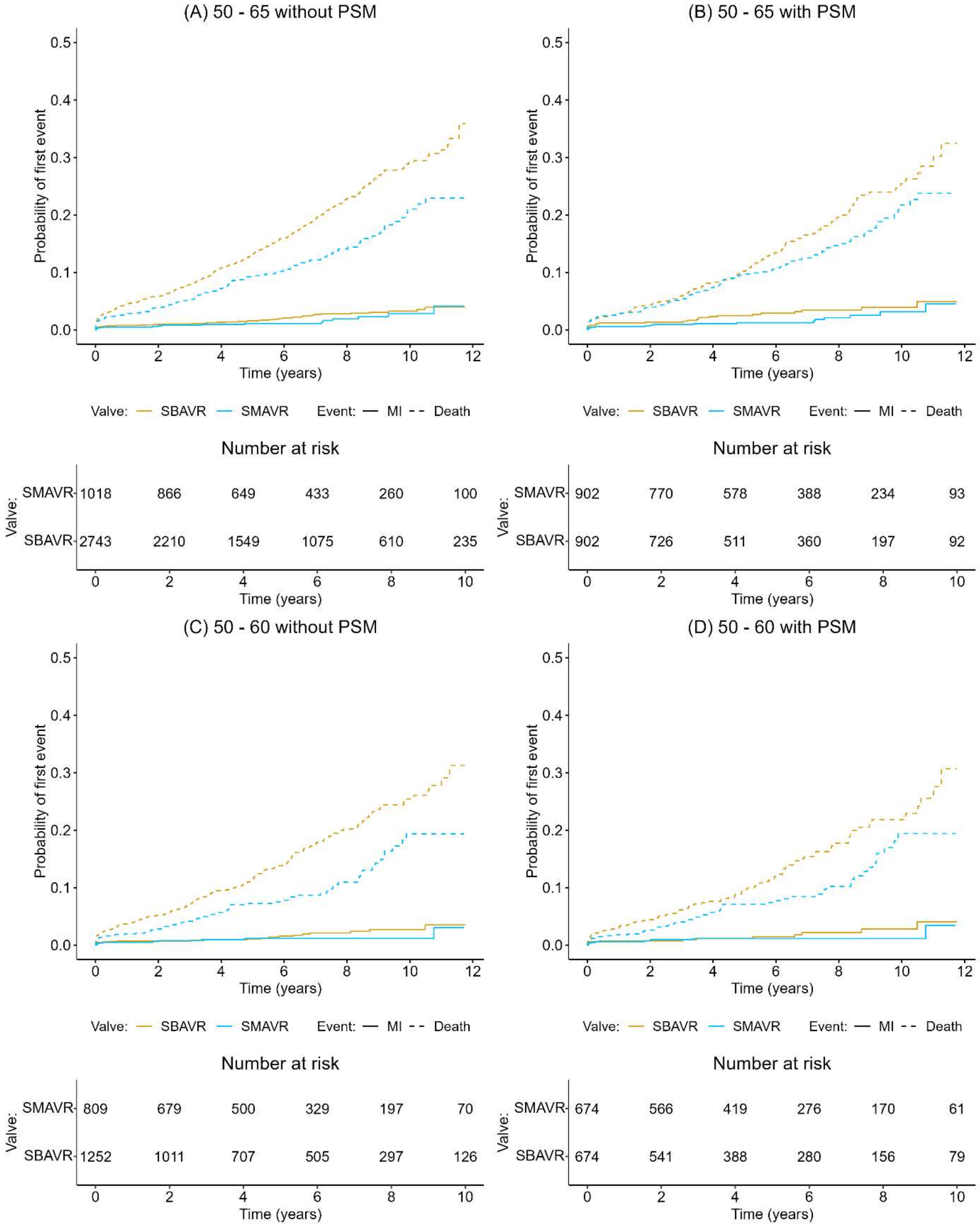

7.6. Embolic stroke or ICH

The following two tables give the number of patients at risk, number of events, number of censored patients as well as the estimated stroke or ICH probability and corresponding 95% confidence intervals for each year up to the 10 years follow up for patients aged 50-65 and the subgroup of patients aged 50-60 before (Table S25) and after (Table S26) propensity score matching.

Table S27 presents the results of the multivariable competing risk models (sub-hazard ratios and corresponding 95% confidence intervals as well as p-values) for patients aged 50-65 and the subgroup of patients aged 50-60 before propensity score matching as well as the results of the multivariable competing risk model with clustering variable matching ID (sub-hazard ratios and corresponding 95% confidence intervals as well as p-values) for patients aged 50-65 and the subgroup of patients aged 50-60 after propensity score matching. From the performed models including interaction terms, only the interaction terms are presented separately.

		Patients aged 50 - 65					Patients aged 50 - 60				
Group	Year	N at risk	N events	N censored	Stroke	95% CI	N at risk	N events	N censored	Stroke	95% CI
SMAVR	0	1018	0	0	0	NA - NA	809	0	0	0	NA-NA
	1	954	15	23	0.015	0.009 - 0.024	761	12	21	0.015	0.008 - 0.025
	2	851	24	107	0.024	0.016 - 0.035	669	17	102	0.022	0.013 - 0.034
	3	755	28	188	0.029	0.020 - 0.041	586	20	173	0.026	0.017 - 0.039
	4	636	34	287	0.037	0.026 - 0.050	491	25	257	0.035	0.023 - 0.050
	5	526	39	381	0.044	0.032 - 0.059	402	27	336	0.039	0.026 - 0.055
	6	423	45	473	0.055	0.040 - 0.073	322	31	410	0.049	0.033 - 0.068
	7	339	48	547	0.062	0.046 - 0.081	263	32	465	0.052	0.035 - 0.072
	8	252	51	623	0.070	0.052 - 0.091	191	34	529	0.059	0.041 - 0.083
	9	164	53	704	0.077	0.057 - 0.100	118	36	596	0.069	0.047 - 0.096
	10	95	53	766	0.077	0.057 - 0.100	66	36	642	0.069	0.047 - 0.096
SBAVR	0	2743	0	0	0	NA - NA	1252	0	0	0	NA -NA
	1	2510	15	23	0.019	0.015 - 0.025	1149	12	21	0.022	0.015 - 0.032
	2	2173	24	107	0.028	0.022 - 0.034	983	17	102	0.037	0.028 - 0.049
	3	1838	28	188	0.036	0.029 - 0.044	833	20	173	0.041	0.031 - 0.054
	4	1492	34	287	0.045	0.037 - 0.053	670	25	257	0.053	0.041 - 0.067
	5	1261	39	381	0.056	0.047 - 0.066	570	27	336	0.064	0.050 - 0.080
	6	1027	45	473	0.062	0.052 - 0.072	474	31	410	0.070	0.055 - 0.087
	7	790	48	547	0.068	0.057 - 0.079	358	32	465	0.075	0.059 - 0.094
	8	577	51	623	0.078	0.066 - 0.092	278	34	529	0.082	0.065 - 0.102
	9	385	53	704	0.084	0.071 - 0.099	188	36	596	0.088	0.069 - 0.110
	10	226	53	766	0.091	0.076 - 0.109	120	36	642	0.098	0.075 - 0.124

Table S25: Number of patients at risk, number of events, number of censored patients as well as the estimated stroke probability and corresponding 95% confidence intervals for patients aged 50-65 and the subgroup of patients aged 50-60.

		PSM matched Patients aged 50 - 65					PSM matched Patients aged 50 - 60				
Group	Year	N at risk	N events	N censored	Stroke	95% CI	N at risk	N events	N censored	Stroke	95% CI
SMAVR	0	902	0	0	0	NA - NA	674	0	0	0	NA - NA
	1	849	13	17	0.014	0.008 - 0.024	635	12	17	0.018	0.010 - 0.030
	2	758	21	91	0.024	0.015 - 0.036	556	17	85	0.026	0.016 - 0.040
	3	672	24	164	0.028	0.018 - 0.040	485	19	146	0.030	0.019 - 0.045
	4	568	30	250	0.037	0.025 - 0.051	411	23	211	0.038	0.025 - 0.055
	5	468	35	334	0.045	0.032 - 0.061	335	25	279	0.043	0.028 - 0.062
	6	379	41	413	0.057	0.041 - 0.077	269	29	339	0.055	0.037 - 0.077
	7	303	44	479	0.065	0.047 - 0.086	221	30	384	0.058	0.039 - 0.082
	8	227	47	544	0.074	0.054 - 0.097	163	32	437	0.067	0.045 - 0.094
	9	150	49	615	0.081	0.059 - 0.107	101	33	493	0.072	0.049 - 0.102
	10	89	49	669	0.081	0.059 - 0.107	56	33	532	0.072	0.049 - 0.102
SBAVR	0	902	0	0	0	NA - NA	674	0	0	0	NA - NA
	1	831	13	17	0.021	0.013 - 0.032	617	12	17	0.027	0.016 - 0.041
	2	712	21	91	0.034	0.024 - 0.048	523	17	85	0.043	0.029 - 0.060
	3	602	24	164	0.038	0.027 - 0.053	447	19	146	0.047	0.032 - 0.065
	4	494	30	250	0.046	0.033 - 0.063	368	23	211	0.058	0.041 - 0.079
	5	420	35	334	0.059	0.044 - 0.078	316	25	279	0.067	0.049 - 0.090
	6	349	41	413	0.063	0.047 - 0.083	264	29	339	0.076	0.055 - 0.101
	7	269	44	479	0.071	0.053 - 0.093	197	30	384	0.083	0.060 - 0.110
	8	191	47	544	0.081	0.060 - 0.105	146	32	437	0.096	0.070 - 0.126
	9	132	49	615	0.085	0.063 - 0.111	109	33	493	0.101	0.074 - 0.133
	10	88	49	669	0.098	0.071 - 0.131	74	33	532	0.118	0.084 - 0.158

Table S26 Number of patients at risk, number of events, number of censored patients as well as the estimated stroke probability for the propensity score matched cohorts of patients aged 50-65 and the subgroup of patients aged 50-60.

	Patients aged 50 – 65 years				Patients aged 50 – 60 years			
	All data		After PSM		All data		After PSM	
Variables	Sub-HR (95% CI)	P-value	Sub-HR (95% CI)	P-value	Sub-HR (95% CI)	P-value	Sub-HR (95% CI)	P-value
Original Model								
Heart valve (SMAVR)	1.139 (0.809 - 1.604)	0.460	1.209 (0.824 - 1.774)	0.330	1.362 (0.907 - 2.045)	0.140	1.516 (0.992 - 2.316)	0.055
Age	1.003 (0.970 - 1.038)	0.860	1.024 (0.981 - 1.070)	0.280	1.036 (0.974 - 1.102)	0.260	1.077 (0.995 - 1.166)	0.068
Sex (M)	1.127 (0.831 - 1.529)	0.440	0.901 (0.567 - 1.431)	0.660	1.186 (0.783 - 1.797)	0.420	1.028 (0.607 - 1.740)	0.920
Heart failure (Yes)	0.617 (0.368 - 1.037)	0.068	0.458 (0.190 - 1.103)	0.082	0.587 (0.267 - 1.293)	0.190	0.463 (0.140 - 1.536)	0.210
Myocardial infarction (Yes)	0.696 (0.281 - 1.720)	0.430	0.325 (0.040 - 2.644)	0.290	0.473 (0.103 - 2.159)	0.330	0.774 (0.093 - 6.423)	0.810
Embolic stroke or ICH (Yes)	3.033 (1.474 - 6.238)	0.003	3.719 (1.149 - 12.036)	0.028	2.918 (1.068 - 7.969)	0.037	2.577 (0.272 - 24.421)	0.410
Diabetes mellitus (Yes)	1.476 (1.048 - 2.079)	0.026	1.569 (0.926 - 2.660)	0.094	1.659 (1.018 - 2.703)	0.042	1.513 (0.781 - 2.931)	0.220
Adiposity (Yes)	1.458 (0.965 - 2.205)	0.074	0.850 (0.405 - 1.786)	0.670	1.470 (0.812 - 2.66)	0.200	1.251 (0.504 - 3.110)	0.630
Hyperlipidemia (Yes)	1.130 (0.815 - 1.568)	0.460	1.421 (0.884 - 2.285)	0.150	1.142 (0.729 - 1.788)	0.560	1.233 (0.712 - 2.135)	0.450
Hyperuricemia/gout (Yes)	1.248 (0.609 - 2.556)	0.550	0.655 (0.214 - 1.999)	0.460	0.336 (0.048 - 2.376)	0.270	0.452 (0.067 - 3.046)	0.410
Valvular, rhythmological, and other CMPs (Yes)	0.953 (0.648 - 1.404)	0.810	1.034 (0.582 - 1.837)	0.910	0.938 (0.557 - 1.579)	0.810	1.131 (0.584 - 2.193)	0.720
Ischemic CMP (Yes)	0.954 (0.705 - 1.291)	0.760	0.888 (0.579 - 1.361)	0.580	1.087 (0.726 - 1.626)	0.690	0.948 (0.593 - 1.515)	0.820
Atherosclerosis (Yes)	1.063 (0.564 - 2.004)	0.850	1.221 (0.457 - 3.266)	0.690	0.454 (0.109 - 1.886)	0.280	0.709 (0.164 - 3.060)	0.640
Pulmonary diseases (Yes)	1.488 (0.728 - 3.042)	0.280	2.710 (1.171 - 6.271)	0.020	1.997 (0.812 - 4.909)	0.130	1.854 (0.645 - 5.326)	0.250
Kidney diseases (Yes)	1.022 (0.585 - 1.787)	0.940	1.234 (0.549 - 2.771)	0.610	1.386 (0.676 - 2.843)	0.370	1.573 (0.645 - 3.837)	0.320
Malignant diseases (Yes)	0.434 (0.135 - 1.397)	0.160	0.578 (0.076 - 4.370)	0.590	0.567 (0.130 - 2.478)	0.450	NA	NA
Interaction Terms								
Heart valve (SMAVR) × Age	0.905 (0.846 - 0.968)	0.004	0.907 (0.835 - 0.985)	0.021	0.871 (0.769 - 0.986)	0.029	0.934 (0.814 - 1.072)	0.330
Heart valve (SMAVR) × Sex (M)	1.012 (0.513 - 1.995)	0.970	0.958 (0.396 - 2.319)	0.920	1.157 (0.483 - 2.770)	0.740	0.646 (0.246 - 1.699)	0.380

Table S27: Sub-Hazard ratios (HRs) and corresponding 95% confidence intervals (CIs) from multivariable competing risk regression models accounting for all listed confounders for embolic stroke or intracerebral hemorrhage (ICH) in all patients and the subgroup of patients aged 50-60 years before and after propensity score matching (PSM)

Figure S8: Cumulative incidence curves for embolic stroke or ICH before (A,C) and after (B,D) PSM for all patients aged 50 – 65 years (A,B) and the subgroup of patients aged 50 – 60 years (C,D)

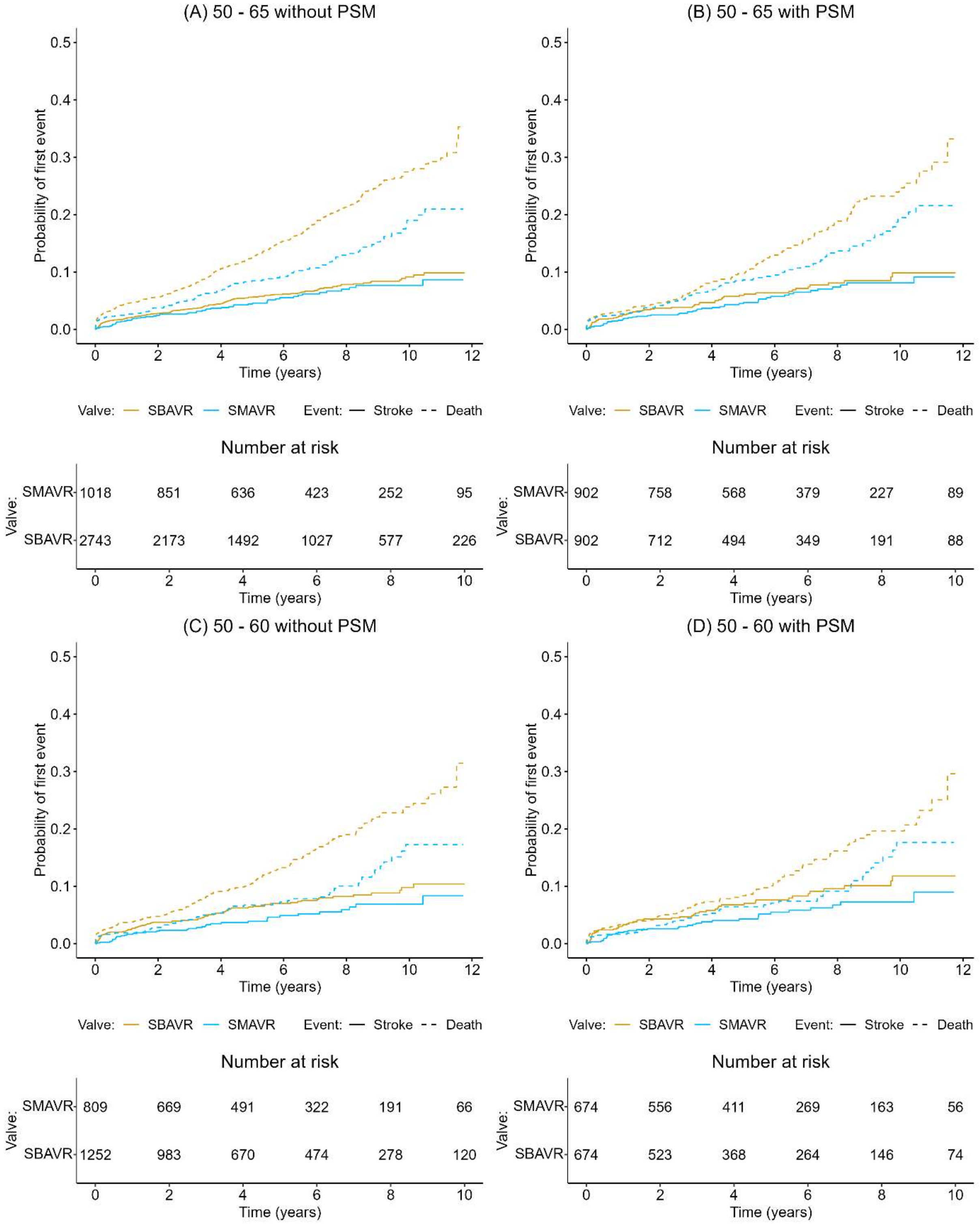

7.7. Bleeding other than embolic stroke or intracerebral hemorrhage

The following two tables gives number of patients at risk, number of events, number of censored patients as well as the estimated bleeding probability and corresponding 95% confidence intervals for each year up to the 10 years follow up for patients aged 50-65 and the subgroup of patients aged 50-60 before (Table S28) and after (Table S29) propensity score matching.

Table S30 presents the results of the multivariable competing risk models (sub-hazard ratios and corresponding 95% confidence intervals as well as p-values) for patients aged 50-65 and the subgroup of patients aged 50-60 before propensity score matching as well as the results of the multivariable competing risk model with clustering variable matching ID (sub-hazard ratios and corresponding 95% confidence intervals as well as p-values) for patients aged 50-65 and the subgroup of patients aged 50-60 after propensity score matching. From the performed models including interaction terms, only the interaction terms are presented separately.

		Patients aged 50 - 65					Patients aged 50 - 60				
Group	Year	N at risk	N events	N censored	Bleeding	95% CI	N at risk	N events	N censored	Bleeding	95% CI
SMAVR	0	1018	0	0	0	NA - NA	809	0	0	0	NA -NA
	1	954	13	23	0.013	0.007 - 0.021	760	12	21	0.015	0.008 - 0.025
	2	852	21	107	0.021	0.014 - 0.032	669	16	102	0.02	0.012 - 0.032
	3	753	25	189	0.026	0.017 - 0.037	583	20	174	0.026	0.017 - 0.039
	4	634	29	291	0.031	0.021 - 0.044	487	24	260	0.033	0.022 - 0.048
	5	520	34	388	0.039	0.027 - 0.053	397	26	340	0.037	0.025 - 0.053
	6	415	40	481	0.049	0.036 - 0.067	315	30	416	0.047	0.032 - 0.066
	7	335	44	551	0.059	0.043 - 0.078	256	33	469	0.056	0.038 - 0.078
	8	254	44	627	0.059	0.043 - 0.078	189	33	532	0.056	0.038 - 0.078
	9	166	47	706	0.071	0.051 - 0.095	117	35	597	0.067	0.045 - 0.094
	10	93	48	771	0.077	0.054 - 0.105	62	36	645	0.076	0.049 - 0.111
SBAVR	0	2743	0	0	0	NA - NA	1252	0	0	0	NA - NA
	1	2528	13	23	0.014	0.010 - 0.019	1163	12	21	0.012	0.007 - 0.019
	2	2187	21	107	0.021	0.016 - 0.027	999	16	102	0.021	0.014 - 0.030
	3	1854	25	189	0.027	0.021 - 0.034	844	20	174	0.029	0.020 - 0.040
	4	1516	29	291	0.033	0.026 - 0.040	689	24	260	0.036	0.026 - 0.048
	5	1291	34	388	0.035	0.028 - 0.043	586	26	340	0.037	0.027 - 0.050
	6	1060	40	481	0.041	0.033 - 0.050	493	30	416	0.040	0.029 - 0.054
	7	817	44	551	0.043	0.035 - 0.053	372	33	469	0.042	0.031 - 0.056
	8	602	44	627	0.044	0.036 - 0.054	289	33	532	0.042	0.031 - 0.056
	9	391	47	706	0.051	0.041 - 0.063	189	35	597	0.045	0.033 - 0.061
	10	227	48	771	0.054	0.043 - 0.067	121	36	645	0.051	0.035 - 0.071

Table S28: Number of patients at risk, number of events, number of censored patients as well as the estimated bleeding probability and corresponding 95% confidence intervals for patients aged 50-65 and the subgroup of patients aged 50-60.

		PSM matched Patients aged 50 - 65					PSM matched Patients aged 50 - 60				
Group	Year	N at risk	N events	N censored	Bleeding	95% CI	N at risk	N events	N censored	Bleeding	95% CI
SMAVR	0	902	0	0	0	NA - NA	674	0	0	0	NA - NA
	1	848	12	17	0.013	0.007 - 0.023	636	10	17	0.015	0.008 - 0.026
	2	758	19	91	0.022	0.013 - 0.033	559	13	85	0.020	0.011 - 0.032
	3	670	23	165	0.027	0.017 - 0.039	485	16	147	0.025	0.015 - 0.040
	4	565	27	253	0.033	0.022 - 0.046	410	20	213	0.033	0.021 - 0.050
	5	464	31	338	0.039	0.027 - 0.055	332	22	283	0.038	0.024 - 0.056
	6	373	36	419	0.049	0.035 - 0.067	263	26	346	0.050	0.033 - 0.072
	7	300	40	481	0.06	0.043 - 0.080	215	29	389	0.061	0.041 - 0.086
	8	229	40	547	0.06	0.043 - 0.080	161	29	440	0.061	0.041 - 0.086
	9	152	43	616	0.073	0.051 - 0.099	100	31	494	0.073	0.048 - 0.105
	10	87	44	673	0.08	0.055 - 0.109	52	32	535	0.085	0.053 - 0.125
SBAVR	0	902	0	0	0	NA - NA	674	0	0	0	NA - NA
	1	840	12	17	0.011	0.006 - 0.020	629	10	17	0.012	0.006 - 0.023
	2	725	19	91	0.015	0.008 - 0.024	536	13	85	0.020	0.011 - 0.033
	3	613	23	165	0.02	0.012 - 0.031	460	16	147	0.022	0.012 - 0.035
	4	506	27	253	0.028	0.018 - 0.041	381	20	213	0.028	0.017 - 0.043
	5	431	31	338	0.028	0.018 - 0.041	324	22	283	0.030	0.019 - 0.047
	6	360	36	419	0.037	0.024 - 0.053	274	26	346	0.036	0.022 - 0.055
	7	278	40	481	0.042	0.028 - 0.059	205	29	389	0.040	0.025 - 0.060
	8	195	40	547	0.042	0.028 - 0.059	153	29	440	0.040	0.025 - 0.060
	9	131	43	616	0.046	0.030 - 0.067	111	31	494	0.040	0.025 - 0.060
	10	87	44	673	0.046	0.030 - 0.067	77	32	535	0.040	0.025 - 0.060

Table S29 Number of patients at risk, number of events, number of censored patients as well as the estimated bleeding probability for the propensity score matched cohorts of patients aged 50-65 and the subgroup of patients aged 50-60.

	Patients aged 50 – 65 years				Patients aged 50 – 60 years			
	All data		After PSM		All data		After PSM	
Variables	Sub-HR (95% CI)	P-value	Sub-HR (95% CI)	P-value	Sub-HR (95% CI)	P-value	Sub-HR (95% CI)	P-value
Original Model								
Heart valve (SMAVR)	0.819 (0.561 - 1.196)	0.300	0.697 (0.439 - 1.106)	0.130	0.825 (0.516 - 1.320)	0.420	0.676 (0.393 - 1.165)	0.160
Age	0.991 (0.952 - 1.033)	0.670	0.999 (0.939 - 1.062)	0.970	0.979 (0.911 - 1.051)	0.550	1.008 (0.912 - 1.115)	0.870
Sex (M)	1.207 (0.842 - 1.730)	0.310	1.015 (0.590 - 1.747)	0.960	1.263 (0.766 - 2.081)	0.360	1.188 (0.635 - 2.221)	0.590
Heart failure (Yes)	1.068 (0.653 - 1.748)	0.790	0.690 (0.276 - 1.725)	0.430	0.884 (0.396 - 1.972)	0.760	0.273 (0.037 - 2.032)	0.210
Myocardial infarction (Yes)	0.819 (0.328 - 2.045)	0.670	0.775 (0.196 - 3.067)	0.720	0.672 (0.157 - 2.868)	0.590	0.925 (0.151 - 5.663)	0.930
Embolic stroke or ICH (Yes)	2.434 (0.950 - 6.237)	0.064	NA	NA	2.764 (0.800 - 9.552)	0.110	NA	NA
Diabetes mellitus (Yes)	1.385 (0.922 - 2.081)	0.120	1.514 (0.801 - 2.863)	0.200	1.012 (0.527 - 1.941)	0.970	0.964 (0.338 - 2.752)	0.950
Adiposity (Yes)	0.858 (0.467 - 1.575)	0.620	0.600 (0.207 - 1.741)	0.350	0.724 (0.290 - 1.812)	0.490	0.486 (0.105 - 2.243)	0.350
Hyperlipidemia (Yes)	0.881 (0.594 - 1.308)	0.530	1.120 (0.643 - 1.95)	0.690	1.113 (0.657 - 1.886)	0.690	1.828 (0.972 - 3.438)	0.061
Hyperuricemia/gout (Yes)	1.913 (0.936 - 3.906)	0.075	1.071 (0.309 - 3.708)	0.910	1.867 (0.640 - 5.448)	0.250	NA	NA
Valvular, rhythmological, and other CMPs (Yes)	1.115 (0.689 - 1.804)	0.660	1.195 (0.572 - 2.497)	0.640	1.411 (0.707 - 2.816)	0.330	1.076 (0.482 - 2.401)	0.860
Ischemic CMP (Yes)	1.265 (0.910 - 1.758)	0.160	1.845 (1.182 - 2.881)	0.007	1.452 (0.932 - 2.264)	0.099	1.214 (0.703 - 2.096)	0.490
Atherosclerosis (Yes)	1.820 (1.023 - 3.237)	0.042	2.511 (1.167 - 5.403)	0.019	1.383 (0.512 - 3.733)	0.520	1.876 (0.677 - 5.199)	0.230
Pulmonary diseases (Yes)	0.611 (0.191 - 1.952)	0.410	NA	NA	NA	NA	NA	NA
Kidney diseases (Yes)	1.552 (0.915 - 2.632)	0.100	0.898 (0.302 - 2.671)	0.850	1.998 (0.999 - 3.994)	0.050	1.619 (0.543 - 4.833)	0.390
Malignant diseases (Yes)	0.939 (0.388 - 2.270)	0.890	2.029 (0.684 - 6.016)	0.200	0.869 (0.228 - 3.308)	0.840	NA	NA
Interaction Terms								
Heart valve (SMAVR) × Age	0.961 (0.885 - 1.043)	0.340	0.954 (0.853 - 1.067)	0.410	0.884 (0.764 - 1.023)	0.098	0.861 (0.725 - 1.024)	0.090
Heart valve (SMAVR) × Sex (M)	2.439 (1.054 - 5.641)	0.037	2.995 (1.057 - 8.493)	0.039	2.892 (1.026 - 8.149)	0.045	2.491 (0.795 - 7.801)	0.120

Table S30: Sub-Hazard ratios (Sub-HRs) and corresponding 95% confidence intervals (CIs) from multivariable competing risk regression models accounting for all listed confounders for bleeding other than embolic stroke or intracerebral hemorrhage (ICH) in all patients and the subgroup of patients aged 50 - 60 years before and after propensity score matching (PSM)

Figure S9: Cumulative incidence curves for bleeding other than embolic stroke or ICH before (A,C) and after (B,D) PSM for all patients aged 50 – 65 years (A,B) and the subgroup of patients aged 50 – 60 years (C,D)

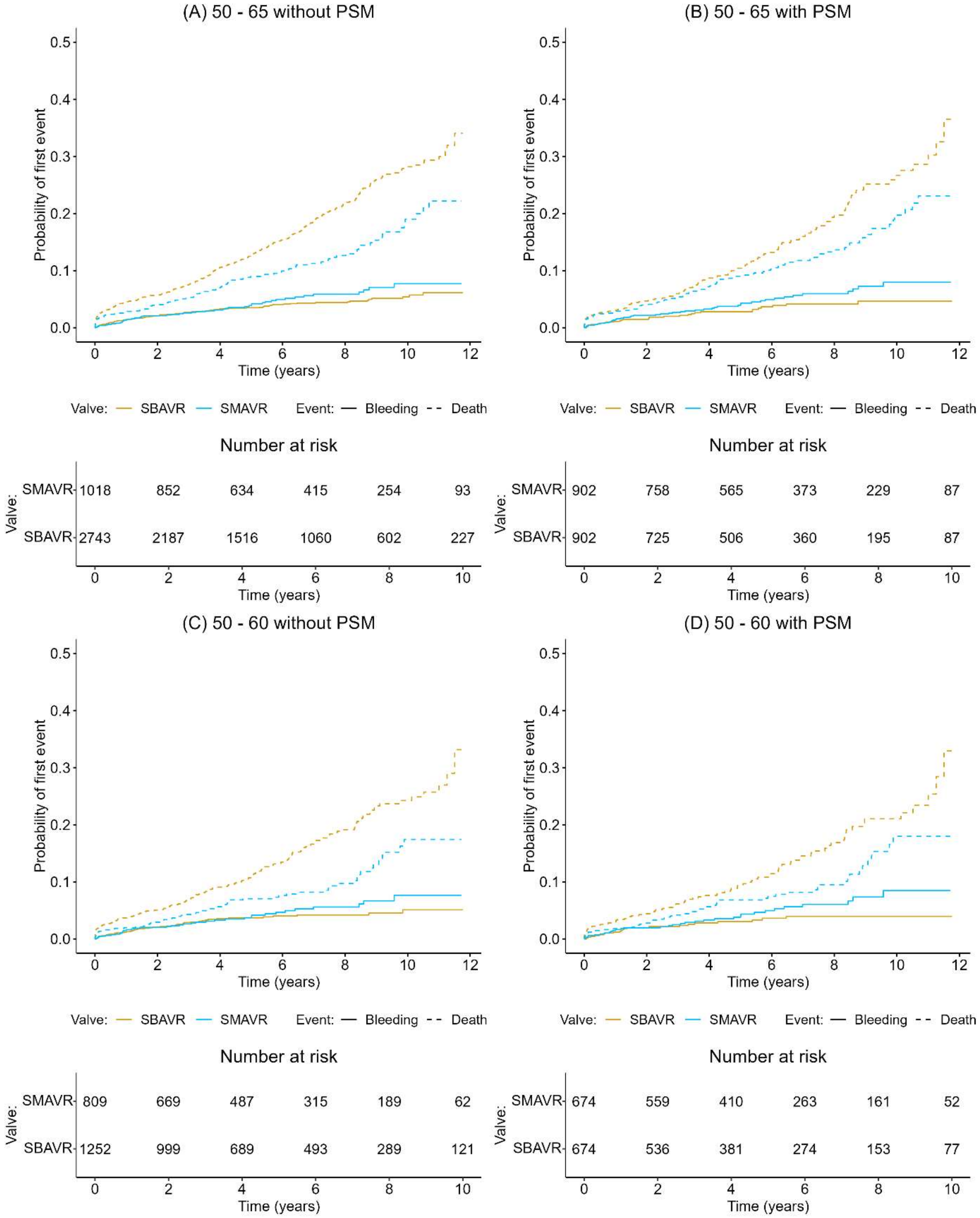

8.) Exploratory Outcome

The following table gives the number of patients at risk, number of events, number of censored patients as well as the estimated survival probability after reoperation and corresponding 95% confidence intervals for each year up to the 10 years follow up for patients aged 50-65 and the subgroup of patients aged 50-60 (Table S31).

		Patients aged 50 - 65					Patients aged 50 - 60				
Group	Year	N at risk	N events	N censored	Survival	95% CI	N at risk	N events	N censored	Survival	95% CI
SMAVR	0	16	0	0	1	1 - 1	14	0	0	1	1 -1
	1	16	0	0	1	1 - 1	14	0	0	1	1 - 1
	2	14	1	1	0.938	0.826 - 1	13	0	1	1	1 - 1
	3	12	0	2	0.938	0.826 - 1	12	0	1	1	1 - 1
	4	9	1	2	0.852	0.680 - 1	9	1	2	0.909	0.754 - 1
	5	7	0	2	0.852	0.680 - 1	7	0	2	0.909	0.754 - 1
	6	7	0	0	0.852	0.680 - 1	7	0	0	0.909	0.754 - 1
	7	5	1	1	0.710	0.465 - 1	5	1	1	0.758	0.506 - 1
	8	3	0	2	0.710	0.465 - 1	3	0	2	0.758	0.506 - 1
	9	1	0	2	0.710	0.465 - 1	1	0	2	0.758	0.506 - 1
	10	1	0	0	0.710	0.465 - 1	1	0	0	0.758	0.506 - 1
SBAVR	0	99	1	0	0.990	0.970 - 1	42	0	0	1	1 - 1
	1	87	11	0	0.879	0.817 - 0.945	39	3	0	0.929	0.854 - 1
	2	71	6	10	0.817	0.745 - 0.897	30	4	5	0.831	0.724 - 0.954
	3	56	1	14	0.805	0.730 - 0.888	26	0	4	0.831	0.724 - 0.954
	4	42	0	14	0.805	0.730 - 0.888	22	0	4	0.831	0.724 - 0.954
	5	30	2	10	0.758	0.666 - 0.862	17	1	4	0.790	0.666 - 0.936
	6	22	1	7	0.733	0.634 - 0.847	14	1	2	0.743	0.604 - 0.915
	7	17	0	5	0.733	0.634 - 0.847	11	0	3	0.743	0.604 - 0.915
	8	8	2	7	0.627	0.483 - 0.814	5	2	4	0.550	0.343 - 0.884
	9	5	0	3	0.627	0.483 - 0.814	4	0	1	0.550	0.343 - 0.884
	10	4	0	1	0.627	0.483 - 0.814	3	0	1	0.550	0.343 - 0.884

Table S31: Number of patients at risk, number of events, number of censored patients as well as the estimated survival probability after reoperation and corresponding 95% confidence intervals for patients aged 50-65 and the subgroup of patients aged 50-60.

Figure S10: Kaplan-Meier curves and 95% confidence intervals for survival after reoperation for all patients aged 50 – 65 years (A) and the subgroup of patients aged 50 – 60 years (B)

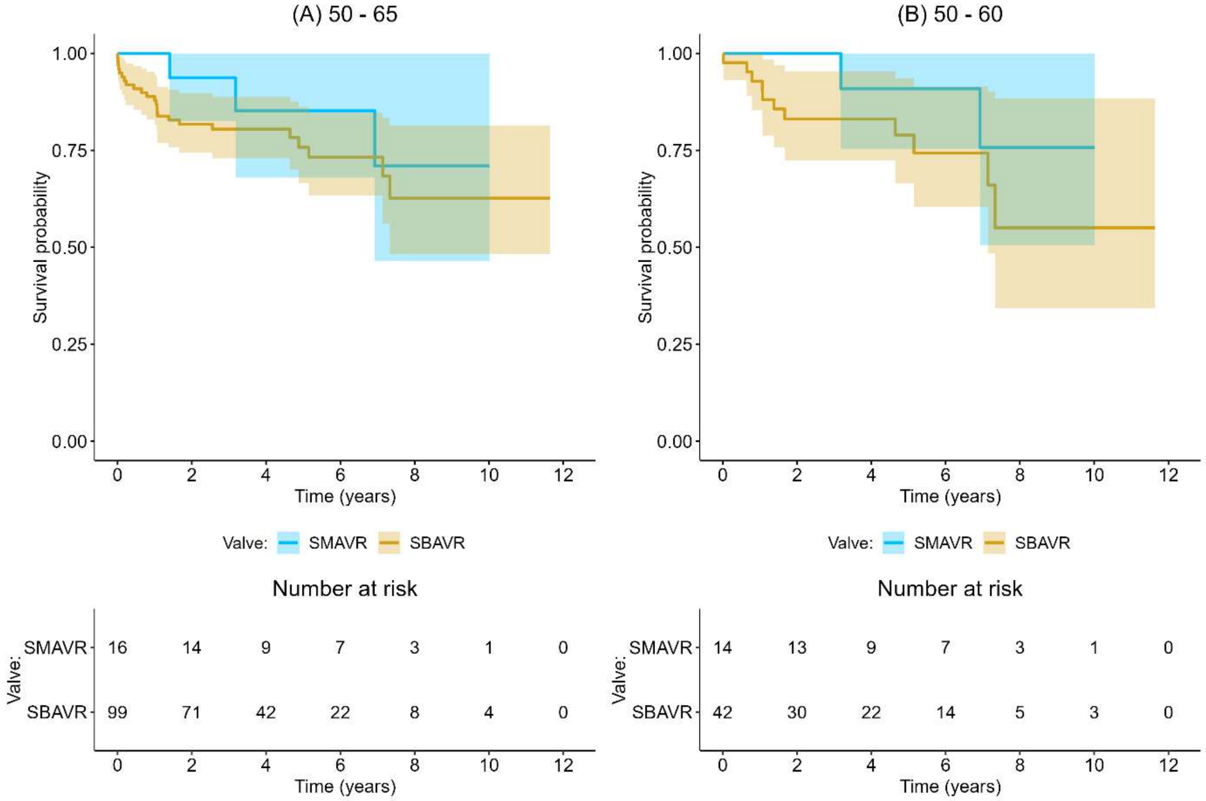

Figure S11: Summary of Hazard ratios (HRs) or sub-hazard ratios (sub-HRs) and 95% confidence intervals from multivariable regression models for all outcomes before (A, C) and after (B,D) PSM for all patients aged 50 – 65 years (A,B) and Patients aged 50 – 60 years (C,D). HR or sub-HRs >1 indicate a higher risk of the event occurring after SBAVR than SMAVR.

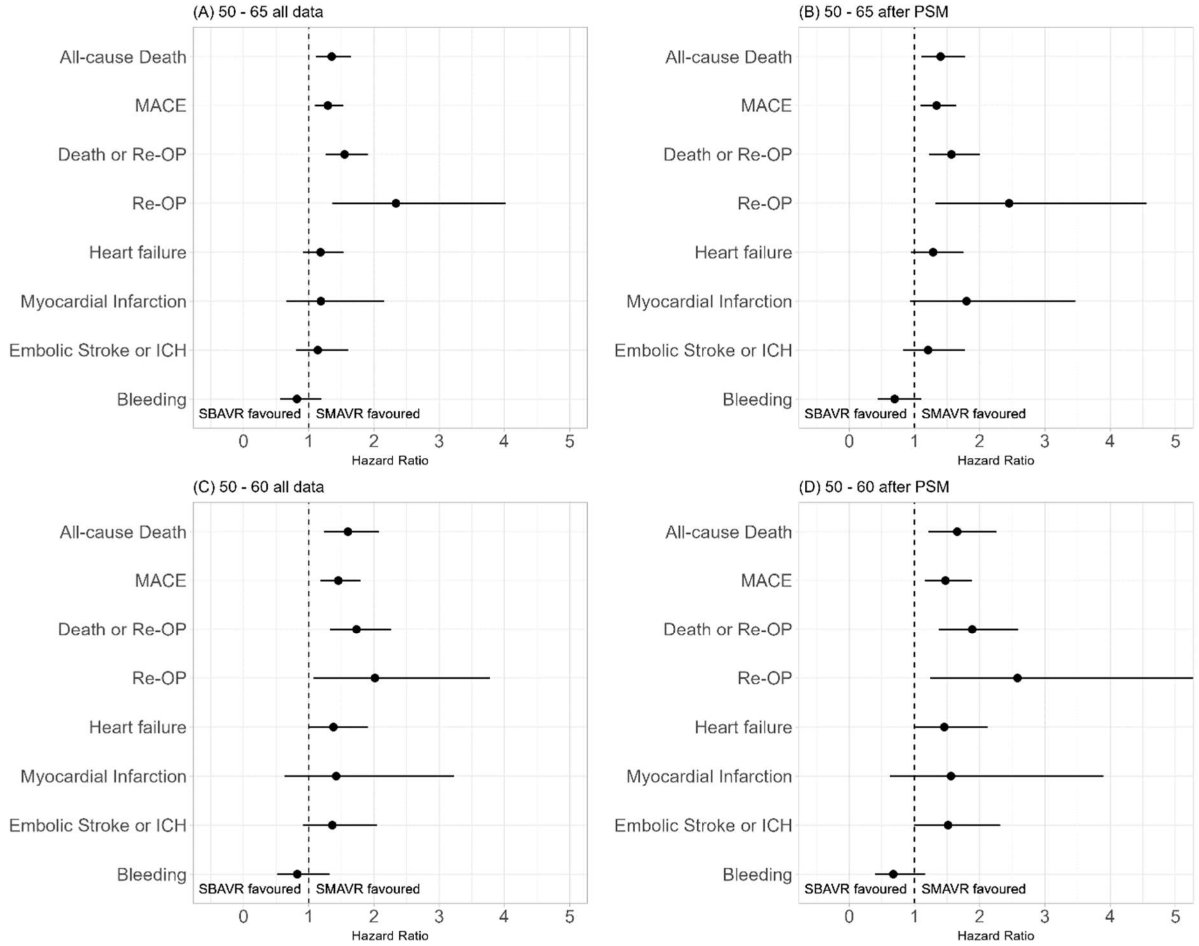

Supplement: ezaf200_Supplementary_Data [file ezaf200_supplementary_data.zip › AUTHEART_IV_Supp_final.pdf]
